# FOXR2 Targets LHX6^+^/DLX^+^ Neural Lineages to Drive Central Nervous System Neuroblastoma

**DOI:** 10.1158/0008-5472.CAN-24-2248

**Published:** 2024-11-04

**Authors:** Selin Jessa, Antonella De Cola, Bhavyaa Chandarana, Michael McNicholas, Steven Hébert, Adam Ptack, Damien Faury, Jessica W. Tsai, Andrey Korshunov, Timothy N. Phoenix, Benjamin Ellezam, David T.W. Jones, Michael D. Taylor, Pratiti Bandopadhayay, Manav Pathania, Nada Jabado, Claudia L. Kleinman

**Affiliations:** 1 Lady Davis Research Institute, Jewish General Hospital, Montreal, Canada.; 2 Quantitative Life Sciences, McGill University, Montreal, Canada.; 3 Department of Oncology, Early Cancer Institute, Adrian Way, University of Cambridge, Cambridge, United Kingdom.; 4 CRUK Children’s Brain Tumour Centre of Excellence, University of Cambridge, Cambridge, United Kingdom.; 5 Department of Human Genetics, McGill University, Montreal, Canada.; 6 Department of Experimental Medicine, McGill University, Montreal, Canada.; 7 Research Institute of the McGill University Health Centre, Montreal, Canada.; 8 Department of Pediatrics, Keck School of Medicine of University of Southern California, Los Angeles, California.; 9 Department of Pediatrics, Cancer and Blood Disease Institute, Children’s Hospital Los Angeles, Los Angeles, California.; 10 The Saban Research Institute, Children’s Hospital Los Angeles, Los Angeles, California.; 11 Clinical Cooperation Unit Neuropathology (B300), German Cancer Research Center (DKFZ), German Cancer Consortium (DKTK), National Center for Tumor Diseases (NCT), Heidelberg, Germany.; 12 Department of Neuropathology, Heidelberg University Hospital, Heidelberg, Germany.; 13 Hopp Children’s Cancer Center Heidelberg (KiTZ), Heidelberg University Hospital and German Cancer Research Center (DKFZ), Heidelberg, Germany.; 14 Division of Pharmaceutical Sciences, James L. Winkle College of Pharmacy, University of Cincinnati, Cincinnati, Ohio.; 15 Department of Pathology, Centre Hospitalier Universitaire Sainte-Justine, Université de Montréal, Montréal, Canada.; 16 Division of Pediatric Glioma Research, Hopp Children’s Cancer Center (KiTZ), Heidelberg, Germany.; 17 National Center for Tumor Diseases (NCT), NCT Heidelberg, a partnership between DKFZ and Heidelberg University Hospital, Heidelberg, Germany.; 18 German Cancer Research Center (DKFZ), Heidelberg, Germany.; 19 Pediatric Neuro-Oncology Research Program, Texas Children’s Hospital, Houston, Texas.; 20 Department of Pediatrics, Hematology/Oncology, Hematology/Oncology Section, Texas Children’s Cancer Center, Baylor College of Medicine, Houston, Texas.; 21 Department of Neurosurgery, Baylor College of Medicine, Houston, Texas.; 22 Department of Laboratory Medicine & Pathobiology, University of Toronto, Toronto, Canada.; 23 Developmental & Stem Cell Biology Program, The Hospital for Sick Children, Toronto, Canada.; 24 The Arthur and Sonia Labatt Brain Tumour Research Centre, The Hospital for Sick Children, Toronto, Canada.; 25 Broad Institute of MIT and Harvard, Cambridge, Massachusetts.; 26 Department of Pediatrics, Harvard Medical School, Boston, Massachusetts.; 27 Dana-Farber Cancer Institute, Boston, Massachusetts.; 28 Boston Children’s Cancer and Blood Disorder Center, Boston, Massachusetts.; 29 Department of Pediatrics, McGill University, Montreal, Canada.

## Abstract

Central nervous system neuroblastoma with *forkhead box R2* (*FOXR2*) activation (NB-FOXR2) is a high-grade tumor of the brain hemispheres and a newly identified molecular entity. Tumors express dual neuronal and glial markers, leading to frequent misdiagnoses, and limited information exists on the role of *FOXR2* in their genesis. To identify their cellular origins, we profiled the transcriptomes of NB-FOXR2 tumors at the bulk and single-cell levels and integrated these profiles with large single-cell references of the normal brain. NB-FOXR2 tumors mapped to *LHX6*^+^/*DLX*^+^ lineages derived from the medial ganglionic eminence, a progenitor domain in the ventral telencephalon. *In vivo* prenatal *Foxr2* targeting to the ganglionic eminences in mice induced postnatal cortical tumors recapitulating human NB-FOXR2–specific molecular signatures. Profiling of FOXR2 binding on chromatin in murine models revealed an association with ETS transcriptional networks, as well as direct binding of FOXR2 at key transcription factors that coordinate initiation of gliogenesis. These data indicate that NB-FOXR2 tumors originate from *LHX6*^+^/*DLX*^+^ interneuron lineages, a lineage of origin distinct from that of other *FOXR2*-driven brain tumors, highlight the susceptibility of ventral telencephalon–derived interneurons to *FOXR2*-driven oncogenesis, and suggest that *FOXR2*-induced activation of glial programs may explain the mixed neuronal and oligodendroglial features in these tumors. More broadly, this work underscores systematic profiling of brain development as an efficient approach to orient oncogenic targeting for *in vivo* modeling, critical for the study of rare tumors and development of therapeutics.

**Significance:**

Profiling the developing brain enabled rationally guided modeling of *FOXR2*-activated CNS neuroblastoma, providing a strategy to overcome the heterogeneous origins of pediatric brain tumors that hamper tumor modeling and therapy development.

*See related commentary by Orr, p. 195*

## Introduction

Central nervous system (CNS) neuroblastoma *forkhead box R2* (*FOXR2*)-activated (NB-FOXR2) is a pediatric brain tumor identified from DNA methylation profiling of a large series of CNS tumors in 2016 (1). Previously classified within primitive neuroectodermal tumors, NB-FOXR2 emerged as a cohesive entity now recognized by the World Health Organization (2), with distinct clinical and molecular features. NB-FOXR2 tumors are supratentorial, occurring primarily in cerebral hemispheres (across cortical lobes), with a mean age-at-diagnosis of 6 years (1) and a slight sex bias with greater prevalence in females (1, 3). Histologically, NB-FOXR2 tumors resemble other undifferentiated CNS tumors with both glial and neuronal compartments, leading to frequent misdiagnosis based on morphology alone (3). By IHC, however, NB-FOXR2 exhibits a unique profile with dual neuronal and oligodendroglial features. They express the neuronal synaptic vesicle protein synaptophysin and the oligodendrocyte lineage transcription factor (TF) OLIG2 (1). Thus, recent studies have used immunopositivity for synaptophysin, ANKRD55, OLIG2, and SOX10; immunonegativity for vimentin; and FISH detection of chromosome 1q gain for their diagnosis (3, 4), along with, whenever possible, DNA methylation profiling (5).

Molecularly, NB-FOXR2 tumors are driven by complex fusions and structural rearrangements that converge on overexpression of the TF *FOXR2* on chromosome X and subsequent expression of *MYC* but not *MYCN*. They also harbor a chromosome 1q gain (1), which has recently been shown to converge on suppression of p53 signaling via *MDM4* overexpression (6). No other recurrent somatic mutations have been identified. *FOXR2* is a pan-cancer oncogene, aberrantly expressed in 70% of adult and pediatric cancer types (7), including diffuse intrinsic pontine gliomas (DIPG), as well as some extracranial neuroblastoma (EC-NB; 8). Whereas *FOXR2* can be activated through structural variations, the predominant regulatory mechanism is through promoter hypomethylation (7). *FOXR2* has been therefore suggested to function similarly to cancer–testis antigen genes, which are located on chromosome X, expressed in the testis, and escape silencing through hypomethylation, leading to aberrant activation in cancers (9).

Although *FOXR2* is a pan-cancer oncogene (7), the unifying molecular and IHC profiles of NB-FOXR2 tumors suggest a shared lineage of origin, as is the case for other brain tumor types, including hemispheric histone 3 G34R/V gliomas (10), midline histone 3 K27M gliomas (11), and subtypes of medulloblastomas (12–14). Indeed, we have shown that these pediatric brain tumors mirror specific, anatomically restricted developmental progenitors, accounting for the locations in which these tumors arise, as well as their associated patterns of genetic alterations (10–14). Importantly, although NB-FOXR2 tumors are designated neuroblastomas due to their dual neuronal–glial presentation, they are clinically and histologically distinct from classic EC-NBs, which arise outside the CNS, are derived from neural crest, and express markers of sympathoadrenal lineages (15).

In this study, we sought to define the cellular context in which NB-FOXR2 occurs and design molecularly faithful tumor models for preclinical targeting. We profiled NB-FOXR2 patient tumor samples by bulk and single-cell transcriptomics and assembled a large single-cell resolution reference of the normal developing telencephalon and postnatal cortex. Combinatorial analysis of patterning TFs and systematic mapping of tumors to the normal brain by machine learning and gene set enrichment approaches show that NB-FOXR2 tumors resemble *LHX6^+^*/*DLX^+^* interneurons derived from the medial ganglionic eminence (MGE), a ventral progenitor domain in the developing telencephalon. We show that targeting *Foxr2* and p53 loss of function (p53^LOF^) to the ventral telencephalon in mice by *in utero* electroporation (IUE) is sufficient to induce brain tumors recapitulating human NB-FOXR2, with transcriptional features of ganglionic eminences (GE)–derived lineages. Profiling of Foxr2 binding sites and chromatin accessibility in murine models revealed an association with ETS transcriptional networks, as well as direct Foxr2 binding at genes encoding key TFs that coordinate initiation of gliogenesis. This confirms that *FOXR2* in the ventral telencephalon can produce neuroblastoma-like brain tumors with dual neuronal/glial phenotype and provides a potential explanation to reconcile their glial programs with a likely origin in interneuron lineages.

## Materials and Methods

### Murine models

#### Vector construction

The piggyBac donor and helper vector system was used to transduce neural stem cells (NSC) *in utero*, as previously described (16). CAG-PBase and PBCAG-GFP were a kind gift from F. Chen and J. LoTurco. CRISPR/Cas9 pX330 vectors containing negative control (5′-GCGACCAATACGCGAACGTC-3′) or Trp53-targeting (5′ACAGCCATCACCTCACTGCA-3′) guide RNA (gRNA) sequences were a kind gift from J. Gronych (17). The piggyBac vectors carrying FOXR2 and Akaluc were cloned into PBCAG-GFP, and their expression was driven by a cytomegalovirus early enhancer/chicken β-actin (CAG) promoter. The piggyBac FOXR2 and Akaluc vectors expressed GFP downstream from a PQR 2A peptide, and FOXR2 was C-terminally tagged with a V5 label upstream from the PQR 2A peptide. The piggyBac and CRISPR vectors used in this study were CAG-PBase, PBCAG-Akaluc-PQR-GFP, PBCAG-FOXR2-V5-PQR-GFP, PX330-p53gRNA-CBh-Cas9, and PX330-controlgRNA-CBh-Cas9.

#### IUE

IUE was performed as previously described (18, 19), with minor modifications for targeting GEs. Timed-mated, pregnant C57BL/6J (RRID: IMSR_JAX:000664) mice were acquired from (Charles River Laboratories) and maintained under pathogen-free conditions in individually ventilated cages, with food and water provided *ad libitum*. All procedures were approved by the University of Cambridge Animal Welfare and Ethical Review Body (AWERB) and carried out under a UK Home Office License (PPL PP2303899) in accordance with the Animals (Scientific Procedures) Act 1986. Pregnant females at E12.5 were anesthetized using 2.5% isoflurane and 1.5 L O_2_/minute, with analgesic support provided preoperatively via subcutaneous delivery of Buprevet at 0.1 mg/kg. Uterine horns were exposed through a 1-cm incision, and individual embryos were digitally manipulated into the correct orientation for intraventricular injection. Pulled borosilicate capillaries were loaded with endotoxin-free DNA and Fast Green dye (0.05%, Sigma) for visualization, and a microinjector (Eppendorf) was used to inject the lateral ventricles with the DNA–dye mixture. Three to five plasmids were injected simultaneously, each up to a final concentration of 2 μg/μL, and 1 to 2 μL of total solution was injected per embryo. DNA was electroporated into GE progenitors using 5-mm tweezertrodes (BTX), applying five square pulses at 35 V, 50 ms each, with 950 ms intervals. The embryos were returned into the abdominal cavity, the muscle and skin were sutured, and the animal was monitored until fully recovered from the procedure.

#### Bioluminescence imaging

Tumor growth was monitored as previously described (19). Tumor-bearing mice received i.p. administration of 100 μL of 15 mmol/L AkaLumine-HCl (HY-112641A, MedChemExpress). Approximately 5 minutes after substrate administration, the mice were anesthetized with 2.5% isoflurane and 1.5 L O_2_/minute, and bioluminescence images were acquired using IVIS Spectrum (PerkinElmer; RRID: SCR_018621). The following conditions were used for image acquisition: open for total bioluminescence, exposure time = 60 seconds, binning = medium: 8, field of view = 13.5 × 13.5 cm, and f/stop = 1. Bioluminescent images were analyzed and exported using Living Image 4.7 software (PerkinElmer; RRID: SCR_014247).

#### *Ex vivo* NSC and gliomasphere isolation and culture

All cell lines used in this study were established from murine tumor models, which were generated as described above. *Ex vivo* cell lines were established as previously described (19). Tumor-bearing C57BL/6J mice were euthanized by CO_2_ exposure. Brains were rapidly dissected in ice-cold dissociation medium containing 20 mmol/L glucose, 81.8 mmol/L Na_2_SO_4_, 30 mmol/L K_2_SO_4_, 5.8 mmol/L MgCl_2_, 250 μmol/L CaCl_2_, 1 mmol/L HEPES, 160 μmol/L NaOH, 0.8 mmol/L kynurenic acid, 50 μmol/L D-APV (D-2-amino-5 phosphonopentanoic acid), 100 U/mL penicillin, 100 μg/mL streptomycin, 5 μg/mL Plasmocin, and 100 μg/mL Primocin. Coronal sections were cut using a brain matrix, and GFP^+^ (tumor) and GFP^-^ regions (stroma) were microdissected under an epifluorescence stereomicroscope (Leica M205, Leica Biosystems). Microdissected tissue was then enzymatically digested into a single-cell suspension using the Papain Dissociation System according to the manufacturer’s instructions (Worthington Biochemical). The dissociated cell solution was separated on an OptiPrep density gradient to remove debris, following which, GFP^+^ cells were sorted using a FACSAria II instrument (BD Biosciences). Sorted cells were plated into NeuroCult NSC proliferation media (STEMCELL Technologies) containing 20 ng/mL EGF (Miltenyi Biotec), 20 ng/mL basic FGF (Miltenyi Biotec), 10 ng/mL platelet-derived growth factor (PDGF)-AA (Shenandoah Biotechnology), 10 ng/mL PDGF-BB (Shenandoah Biotechnology) and 2 μg/mL heparin (STEMCELL Technologies). Cells were grown as spheroids (neurospheres) using ultralow-attachment plates (Corning). Cell lines were authenticated using short tandem repeat profiling, and contamination with *Mycoplasma* was tested using qPCR-based PhoenixDX Mycoplasma Detection Kit (last test date February 14, 2023). The results were recorded with the cell line authentication service at the CRUK Cambridge Institute. Cell lines at passage number 12 to 16 were used in experiments.

#### RNA extraction and qPCR

RNA was extracted using RNeasy Plus kit (Qiagen) according to the manufacturer’s instructions. RNA quality and quantification was carried out using a NanoDrop spectrophotometer (Thermo Fisher Scientific). A measure of 500 ng of total RNA was used for reverse transcription using TaqMan Reverse Transcription Reagents (Thermo Fisher Scientific), and RT-PCR was performed using SsoAdvanced Universal SYBR Green Supermix (Bio-Rad) on the CFX96 Touch RT-PCR Detection System (Bio-Rad; RRID: SCR_018064). Primers are described in Supplementary Table S1. The 2^−ΔΔCT^ method was used to calculate relative gene expression levels, and gene expression was normalized to mouse β-2-microglobulin (*B2m)* levels.

#### Immunofluorescence *in vivo*

For immunofluorescence, free-floating sections were incubated in a blocking solution (10% goat or donkey serum, 3% bovine serum albumin (BSA), 0.3% Triton-X in PBS for 1 hour at room temperature and then incubated with primary antibodies at 4°C overnight (Supplementary Table S1). The sections were washed in PBS-Tween (0.05%) before addition of Hoechst 33342 and Alexa Fluor–conjugated secondary antibodies in blocking solution for 1 hour at room temperature. Following washing, the sections were mounted in ProLong Diamond Antifade Mountant (Thermo Fisher Scientific) and imaged using a confocal microscope (Leica SP8, Leica Biosystems).

#### Orthotopic allotransplantation

Orthotopic allotransplantation was performed as previously described (19). Eight-week-old male C57BL/6J mice (Charles River Laboratories) were maintained under pathogen-free conditions in individually ventilated cages, with food and water provided *ad libitum*. All procedures were approved by the University of Cambridge AWERB and carried out under a UK Home Office License (PPL PP2303899) in accordance with the Animals (Scientific Procedures) Act 1986. Analgesia was provided preoperatively via a s.c. injection of Buprevet (0.1 mg/kg). The mice were anesthetized (2.5% isoflurane and 1.5 L O_2_/minute), and the heads of the mice were fixed in a stereotactic frame (#51730, Stoelting Europe). A midline incision was made along the scalp to expose the skull, and a small burr hole was made using a high-speed drill at defined stereotaxic coordinates, 0.5 mm anterior and 1.8 mm lateral from the bregma, to target the striatum. A total of 1.5 × 10^5^*Foxr2* p53 loss-of-function (p53^LOF^) cells resuspended in 5 μL of PBS were then delivered at a depth of 3.2 mm using a 26-gauge (2 mm, AS point style) Hamilton syringe at a controlled rate of 2 μL/minute before the needle was then removed at a rate of 0.5 mm/minute. The scalp was then closed with sutures, and the mice were placed in a heat chamber until fully recovered before being returned to their home cage.

### Library preparation and sequencing

#### Bulk RNA sequencing

##### Human tumors

Total RNA was extracted from cell pellets using AllPrep DNA/RNA/miRNA Universal Kit (Qiagen) according to instructions from the manufacturer. Library preparation was performed with ribosomal RNA (rRNA) depletion according to instructions from the manufacturer (Epicenter). Paired-end sequencing (100 bp) was performed on the Illumina HiSeq 4000 platform.

##### Murine cell lines

Total RNA was extracted from cell pellets using Maxwell RSC simplyRNA Cells Kit (AS1390, Promega) according to instructions from the manufacturer. Library preparation was performed with rRNA depletion according to instructions from the manufacturer (New England Biolabs). Paired-end sequencing (100 bp) was performed on the Illumina NovaSeq6000 S4 v1.5 platform.

#### Single-nuclei RNA sequencing

Nuclei were prepared as previously described (20) from frozen tissue as follows. Frozen tissues (5–50 mg) were dounced on ice in 3 mL of lysis buffer (10 mmol/L Tris-HCl, pH 7.4, 10 mmol/L NaCl, 3 mmol/L MgCl_2_, and 0.05% NP-40; 5 times with a “tight” pestle and then 10 times with a “loose” pestle). Two mL of chilled lysis buffer were then added, and samples were incubated for 5 minutes on ice. Five mL of Nuclei Wash and Resuspension Buffer (NWRB: PBS, 5% BSA, 40 U/mL RNase inhibitor, and 0.25% glycerol) were then added, and nuclei suspensions were passed through a 30-μm cell strainer to remove clumps and centrifuged (500 *g* for 5 minutes). Nuclei pellets were washed with 5 mL of NWRB and centrifuged again. Nuclei pellets were resuspended in a final volume of 1 mL of NWRB, 1 mL of OptiPrep 50% (OptiPrep + solution B :150 mmol/L KCl, 5 mmol/L MgCl_2_, and 20 mmol/L Tricine, pH 7.8, v/v) was added. This 25% OptiPrep solution was layered on 29% OptiPrep cushion and centrifuged at 10,000 *g* for 30 minutes at +4°C. For 10X Genomics 3′RNA protocol, the nuclei pellet was carefully resuspended in NWRB to reach a concentration of 1,000 nuclei/μL. Nuclei concentration was assessed using the ReadyProbes Cell Viability fluorescence assay (Thermo Fisher Scientific). A total of 10,000 to 20,000 nuclei per sample were loaded on the Chromium Controller. Cell capture and library preparation were performed according to the Chromium Single Cell 3′ (v3) protocol for snRNA sequencing (snRNA-seq). The 10x libraries were then sequenced (multiplexed) on the Illumina HiSeq4000 or NovaSeq sequencing platform.

#### Joint single-nuclei RNA and chromatin accessibility profiling (Multiome)

##### Human tumors

Cell nuclei were prepared using automated nuclei preparation following the GentleMACS Octo dissociator protocol (Miltenyi Biotec). Frozen tissue was directly transferred to a precooled gentleMACS “C” tube containing 2 mL of cold Nuclei Extraction Buffer (Miltenyi Biotech) and RNase inhibitor (Protector RNase Inhibitor, Roche). Protocol 4C_nuclei_1 was then run on the gentleMACS Octo dissociator (Miltenyi Biotech). After the run, the sample was incubated for 5 minutes on ice. Nuclei suspension was then applied on the top of a 70-μm SmartStrainer. After centrifugation (5 minutes at 400 *g* for 5 minutes), the pellet was resuspended in NWRB (2% BSA and 0.25% glycerol in PBS) containing RNase inhibitor. Nuclei suspension was then applied on the top of a 30-μm SmartStrainer and centrifuged again. The pellet was resuspended in NWRB, and nuclei were counted using ReadyProbes Cell Viability Blue/Green Kit (Invitrogen). Nuclei were washed one time in diluted nuclei buffer (DNB; 10X Genomics) and resuspended in fresh DNB buffer before loading 20,000 nuclei per sample in the 10X Chromium Controller (10X Genomics). Library preparation (Next GEM Single Cell Multiome) was performed following the manufacturer’s instructions, and sequencing was done on the Illumina HiSeq4000 or NovaSeq sequencing platform.

##### Mouse models

Cell nuclei were prepared using automated nuclei preparation following the Singulator S100 protocol (S2 Genomics). Nuclei were isolated using a nuclei isolation kit and the Singulator S100 instrument from S2 Genomics. Briefly, 5 to 20 mg of frozen tissue were put in a precooled nuclei isolation cartridge with RNAse inhibitors. Samples were then processed on the Singulator S100 instrument following the “extended nuclei” protocol. After nuclei preparation, nuclei were centrifuged and washed twice in Diluted Nuclei Buffer (10x Genomics) and counted with ReadyProbes Cell Viability Blue/Green Kit (Thermo Fisher Scientific). A total of 20,000 nuclei/sample were loaded on the Chromium Controller (10x Genomics). Library preparation (Next GEM Single Cell Multiome) was performed following the manufacturer’s instructions, and sequencing was done on the Illumina HiSeq4000 or NovaSeq sequencing platform.

#### Chromatin immunocleavage sequencing (Cleavage Under Targets and Release Using Nuclease)

The *Foxr2 p53*^LOF^ murine cell line was used for Cleavage Under Targets and Release Using Nuclease (CUT&RUN) chromatin immunocleavage sequencing (*n* = 2 replicates) with antibody against the V5 tag, which was included in the *Foxr2 p53*^LOF^ IUE vector. As a control for the V5 tag, cell lines from a separate IUE mouse model lacking the V5 tag was also subjected to CUT&RUN with the same protocol (*n* = 2 replicates).

Reagents and protocol were based on the EpiCypher CUT&RUN commercial protocol. Prior to the protocol, cross-linking was performed in suspension with 0.1% formaldehyde for 1 minute, followed by reverse cross-linking with glycine, according to the EpiCypher CUTANA cross-linking protocol. Briefly, 5 × 10^5^ cells per sample were dissociated, washed, and bound to CUTANA concanavalin A–coated paramagnetic beads (EpiCypher). The V5 tag antibody (D3H8Q, CST 13202, RRID: AB_2687461) was bound to cells overnight at a 1:50 dilution. Digestion of target chromatin used CUTANA pAG-MNase, followed by DNA collection. Libraries were generated using Kapa HTP Illumina library preparation reagents using 9 to 12 cycles of PCR, followed by dual 0.6 to 0.8× size selection using AMPure XP magnetic beads. Libraries were sequenced on the Illumina NovaSeq6000 platform for approximately 30 million reads per library.

### Bulk RNA-seq

#### Data processing and quality control

External datasets of EC-NB [Gartlgruber and colleagues (21) and Therapeutically Applicable Research to Generate Effective Treatments (TARGET)] were processed as follows. The Gartlgruber 2021 (21) dataset was downloaded as count matrices as provided by the authors (https://nbseb087.dkfz.de/project_NB_SE_viz/, July 2022). Data from the TARGET neuroblastoma study (https://gdc.cancer.gov/content/target-nbl-publication-summary) were downloaded as count matrices from the public Genomic Data Commons cancer portal (https://portal.gdc.cancer.gov/) using Cohort Builder with the following options: project = TARGET NBL, experimental strategy = RNA-seq, and access = open. Normalization (mean of ratios) was performed using DESeq2 (v1.14.1, RRID: SCR_015687; ref. 22), separately for each dataset.

All other samples (Supplementary Table S2 RNAseq column) were processed as follows. Adapter sequences and the first four nucleotides of each read were removed from the read sets using Trimmomatic (v0.32 for human tumors and v0.39 for murine cell lines, RRID: SCR_011848; ref. 23). Reads were scanned from the 5′ end and truncated when the average quality of a four-nucleotide sliding window fell below a threshold (phred33 < 30). Short reads after trimming (<30 base pairs) were discarded. High-quality reads were aligned to the reference genome (human: GRCh37; mouse: mm10) using STAR (RRID: SCR_004463; ref. 24) with default parameters (v2.3.0e for human and v2.7.9a for mouse samples), discarding reads mapping to more than 10 locations [mapping quality (MAPQ) <1]. BigWig files for visualization were created by first generating a bedgraph file using the genomeCoverageBed function from bedtools (v2.30.0, RRID: SCR_006646; ref. 25), with the split and bg parameters, normalized by coverage (number of mapped reads in the sample divided by 100 million). Next, the bedGraphToBigWig function from UCSC tools (v387; ref. 26) was used to create final BigWig files. Quality control (QC) metrics per sample are reported in Supplementary Table S3.

#### Gene expression estimation and differential expression analysis

Gene expression levels were estimated by quantifying primary alignments mapping to at most two locations (MAPQ ≥3) to exonic regions defined by ensGene annotation set from Ensembl (GRCh37, *N* = 60,234 genes) using featureCounts (v1.4.4, RRID: SCR_012919; ref. 27). Normalization (mean of ratios), variance-stabilized transformation of the data, and differential gene expression analysis were performed using DESeq2 (v1.14.1, RRID: SCR_015687; ref. 22).

### Single-cell datasets

#### Read alignment and demultiplexing, cell detection, and count estimation

Single-cell RNA-seq (scRNA-seq)/snRNA-seq datasets (Supplementary Table S2 sc/snRNA-seq column) were processed with Cell Ranger (10x Genomics, v2.0.0, RRID: SCR_017344, “count” option with default parameters) to filter and align sequencing reads to the genome, distinguish cells from background, and obtain gene counts per cell. The hg19 reference genome build, coupled with the Ensembl transcriptome (v75), was used for alignment, including reads mapping to intronic regions.

Multiome datasets (Supplementary Table S2 scMultiome column) were processed with Cell Ranger ARC (10x Genomics, v2.0.0, RRID: SCR_023897, “count” option with default parameters) to filter and align reads, identify transposase cut sites, detect accessible chromatin peaks, call cells, and generate raw count matrices. Human samples were aligned to the hg19 genome, coupled with the Gencode v28 (Ensembl 92) gene annotation. Mouse samples were aligned to a custom mm10 reference integrating the different vectors added to the mouse genome. Five custom chromosomes were added to the mm10 genome, containing (i) the piggyBac transferase; (ii) Akaluc and the *GFP* sequences; (iii) the human *H3F3A* gene with the HA tag; (iv) the p53 guide RNA; and (v) the shATRX sequence. A custom GTF file was created to count the reads mapping to these added sequences.

#### QC and normalization

QC metrics were computed separately for the RNA and assay for transposase-accessible chromatin (ATAC) modalities. Cells were filtered first based on the QC metrics for the RNA modality: mitochondrial content (indicative of cell damage), number of genes, and number of unique molecular identifiers. In the case of Multiome samples, cells were subsequently filtered based on the following ATAC metrics: total number of fragments in peaks and total number of transposition sites across peaks calculated by Cell Ranger ARC, transcription start site enrichment score, and nucleosome signal calculated with Signac (v1.3.0, RRID: SCR_021158; ref. 28). Specific thresholds for each sample, based on the distribution of each metric in the sample, are specified in Supplementary Tables S4 and S5.

RNA libraries were next scaled to 10,000 unique molecular identifiers per cell and log-normalized. QC and downstream data processing was performed using the Seurat and Signac packages [Seurat (29) v3.2.1, RRID: SCR_016341, for sc/snRNA-seq and Signac (28) v1.3.0, RRID: SCR_021158, with Seurat (29) v4.0.0 for Multiome]. Cell-cycle scores for G2/M and S phases were obtained as implemented in Seurat (v3.2.1; ref. 29), by calculating the average expression of G2/M and S phase–associated gene lists in each single cell and subtracting the average expression of control gene lists. Control gene lists were derived by binning genes in each input list into 24 bins according to expression levels and randomly selecting 100 control genes from within each expression bin.

#### Clustering and dimensionality reduction

Counts were *z*-scored gene-wise, and dimensionality reduction was performed using principal component analysis applied to the top 2,000 most variant genes. The first 30 principal components (PC) were then used as input for projection to two dimensions, using uniform manifold approximation and projection (arXiv:1802.03426), and for clustering, using a shared nearest neighbor modularity optimization algorithm based on the Louvain algorithm on a *k*-nearest neighbors graph with *k* = 20, random seed 100, and clustering resolution 0.5.

#### Doublet cell detection

Doublet removal was performed separately for each sample. Cells were clustered (30 PCs, resolution 0.2, and random seed 42), and scDblFinder (v1.8.0, RRID: SCR_022700; ref. 30) was used to detect doublet cells (parameter method = “griffiths,” threshold: *P* value < 0.1). Doublet cells were removed, and clusters with fewer than 20 cells remaining were considered doublets and also removed from downstream analyses.

#### Machine learning–based cell type annotation

Labeling of cells was performed at the individual cell level using an automated, reference-based annotation workflow that combines four prediction methods, detailed below. Briefly, methods were trained on two murine forebrain single-cell references, covering developmental (11) and adult (31) stages (Supplementary Table S6). Cell types were aggregated into broad labels following an ontology provided in Supplementary Table S7, and a consensus annotation was assigned when at least two methods agreed. Cells with no majority or with ties between methods were labeled as “no consensus.”

##### Prediction tools

Four prediction tools were used with default parameters unless specified: SciBet (32), SingleCellNet (33), a correlation-based approach, and support vector machines (SVM; ref. 34). SciBet (v1.0, RRID: SCR_024743) selects marker genes and assigns cells to their respective cell types using multimodal distribution models and maximum likelihood estimation. SingleCellNet (v0.1.0, RRID: SCR_024742) is based on selection of most discriminative pairs of genes for each cell type to train a multi-class Random Forest classifier. Training was run with 500 trees (nTrees,) and prediction was run with 0 randomized cell profiles (nrand). In the correlation-based approach, the Spearman correlation coefficient is calculated between the query cell expression matrix and mean expression matrix of each reference cell type, and the cell type label with the maximum coefficient is selected. Finally, a linear SVM (34) was implemented through sklearn LinearSVC (v1.3.0, RRID: SCR_024741) and trained on the reference dataset using cross-validation to estimate model parameters (regularization parameter and loss function).

##### Training datasets and consensus calculations

All methods were trained on the intersection of genes detected in both the reference and query datasets, with intersections computed separately for mouse and human predictions.

For human tumor cell annotation, a forebrain single-cell developmental reference (11) was used for training, with gene symbols converted to human gene symbols using the R packages Orthology.eg.db (v3.16, RRID: SCR_024740, https://doi.org/doi:10.18129/B9.bioc.Orthology.eg.db), org.Mm.eg.db (v3.16, RRID: SCR_023488, https://doi.org/doi:10.18129/B9.bioc.org.Mm.eg.db), and org.Hs.eg.db (v3.16, RRID: SCR_024739, https://doi.org/doi:10.18129/B9.bioc.org.Hs.eg.db). For mouse tumor cells, in turn, in which no cross-species prediction is performed and thus higher resolution is feasible, annotation was performed in two steps. First, cells were annotated with models trained on the developing mouse forebrain reference (11). Next, cells labeled as neurons were reannotated with models trained on a postnatal mouse brain reference with high neuronal diversity. This forebrain reference was constructed from a large (*n* = 4 million cells) adult murine cell atlas (31) by (i) subsetting forebrain regions, i.e., cells annotated as isocortex, olfactory areas, cortical subplate, or cerebral nuclei by the authors, (ii) removing any cell types (subclass level defined by the authors) containing fewer than 100 cells, and (iii) randomly subsampling each cell type to contain a maximum of 500 cells; the final training dataset contained *n* = 52,991 cells. Predicted labels from each method were aggregated into broad cell classes defined in the original study (also detailed in Supplementary Table S7) before computing a consensus. We then categorized each resulting label as neuron, glia, or other (Supplementary Table S7) and labeled any cell in nonneuronal categories (i.e., with labels discrepant between the two references) as “no consensus.”

Training and consensus predictions were performed using the automated pipeline CoRAL (v3.0.0, https://github.com/fungenomics/CoRAL).

#### Copy number variation inference and malignant cell calling

##### Human tumors

Copy-number variation (CNV) profiles were inferred for each sample using inferCNV (v1.7.2, RRID: SCR_021140, https://github.com/broadinstitute/infercnv), with the following parameters: window_length = 101 genes, cutoff = 0.1, analysis_mode = “samples,” cluster_by_groups = FALSE, and denoise = TRUE). The hg19 Gencode v19 was used as gene annotation, excluding mitochondrial genes (defined as gene symbols starting with “MT-”), ribosomal genes (defined as gene symbols starting with “MRPS,” “MRPL,” “RPS,” and “RPL”), and HLA genes (defined as gene symbols starting with “HLA-”). Normal cells extracted from a published collection of high-grade gliomas (HGG; ref. 10) were used as a reference. Hierarchical clustering of cells based on their CNV profiles allowed identification of subtrees of cells with clear CNV signal, which were labeled as malignant, and subtrees with no defined CNVs, which were labeled as normal. All samples were next combined for a joint clustering analysis, and clusters containing multiple samples and more than 5% normal cells based on inferCNV were also labeled as normal.

##### Mouse models

InferCNV analysis was performed using as reference a normal postnatal mouse brain cortex sample (35), augmented with normal cells from the murine samples in this study, defined as immune cells (through machine learning–based annotation) and clusters of cells with low *Foxr2* expression. These clusters were identified as follows. A neighbor graph was constructed for each sample using the first 20 PCs as input, and a first round of clustering was performed as for doublet cell detection. Next, cells within each cluster were reclustered (random seed 100, resolution 0.2), excluding any cells labeled as immune cells; subclusters with fewer than 10 cells were discarded. For each subcluster, a differential expression test (Wilcoxon rank-sum) of *Foxr2* expression was performed between the subcluster and the immune population using the Seurat (v4.0.0; ref. 29) package function FindMarkers. Subclusters in which *Foxr2* expression was not significantly higher than in immune cells (*P* value ≥ 0.05) were considered to contain normal cells and added to the reference. Finally, to refine the normal reference, inferCNV (v1.7.2, RRID: SCR_021140) was run using the same cells as reference and query, and subtrees of cells containing CNVs were removed.

Using this reference of normal cells, CNV profiles were inferred for each sample using inferCNV with the following parameters: window_length = 101 genes, cutoff = 0.1, analysis_mode = “samples,” cluster_by_groups = FALSE, sd_amplifier = 1.75, and denoise = TRUE. The GRCm38 Ensembl genome v84 was used as gene annotation with the following modifications. The mitochondrial genes (defined as having gene symbols starting with “mt-”), ribosomal genes (defined as having gene symbols starting with “Mrps,” “Mrpl,” “Rps,” and “Rpl”), MHC genes (defined as having gene symbols starting with “H2-” and “H60”), and any chromosome other than 1 to 19, X, or Y were excluded from the annotation.

#### Generation of pseudobulk ATAC genomic tracks by cell type

To visualize genomic tracks for ATAC signal in Multiome samples, pseudobulk BigWig files were generated for cells in each annotated cell type by subsetting the BAM file containing all aligned reads from the ATAC modality of that sample. The subset_bam (v1.1, https://github.com/10XGenomics/subset-bam) tool was run with the following input: cell barcodes for each group of cells and the full-sample ATAC BAM file produced from Multiome processing with CellRanger (described earlier). Subsetted BAM files were indexed using Samtools (v1.17, RRID: SCR_002105; ref. 36), and BigWig tracks were produced using deepTools (v3.5.0, RRID: SCR_016366; ref. 37) with counts per million (CPM) normalization, removing the mitochondrial chromosome.

### Cross-species integrative analysis

#### TF fingerprint and SVM analysis

scRNA-seq data were retrieved from five published datasets (Supplementary Table S6) that profiled human 9 to 12 postconceptional week (PCW) GEs (denoted as fetal human 1; ref. 38), human 9 to 18 PCW GEs (fetal human 2; ref. 39), mouse E10-P6 forebrain (fetal mouse; refs. 11, 12), adult mouse isocortex (adult mouse; ref. 40), and adult human cortical medial temporal gyrus (adult human; ref. 41). For each dataset, we obtained the single-cell gene counts and the cluster cell type labels as reported in the original studies. Counts for each cell were scaled to a total of 10,000 per cell and log-normalized using Seurat (v4.0.0; ref. 29). Clusters with fewer than 20 cells were excluded from the analysis.

To harmonize cell type labels across the five datasets, the labels reported in the original studies were used to assign each cluster into one of seven classes representing telencephalon-derived populations: caudal GE/lateral GE (CGE/LGE)-derived, MGE-derived, excitatory neurons, other neurons, progenitors, glia, and nonneuroectodermal, as follows. For human developmental datasets, authors of the original studies had indicated in the labels the MGE, CGE, LGE, or dorsal/excitatory origin of each cluster. For the mouse embryonal dataset, MGE and excitatory labels were used from the original dataset, and other interneurons, including “cortical inhibitory neurons” and “striatal spiny neurons” were considered CGE/LGE-derived (42). For mouse and adult human datasets, the classification schemes from the original studies were used (40, 41), in which inhibitory neuron clusters labeled as parvalbumin^+ ^(PVALB) and somatostatin (SST) subtypes were considered MGE-derived, inhibitory neuron clusters labeled as VIP, LAMP5, or PAX6 subtypes were labeled as CGE/LGE-derived; and excitatory neurons were labeled as excitatory. Any neuron clusters that could not be confidently included in one of these categories were considered “other neurons.” Only clusters in the CGE/LGE-derived, MGE-derived, and excitatory classes were retained for the remainder of the fingerprint analysis (Supplementary Table S8).

For each dataset, TF expression was treated as follows. First, a common gene feature space across mouse and human datasets was defined by replacing mouse orthologs with their one-to-one human orthologs. Next, the mean of the log-normalized expression was calculated for each TF in each cluster and scaled to (0, 1) across clusters. This scaling was performed within each dataset because different datasets may not have directly comparable expression ranges. Second, the detection rate of each TF was calculated in each cluster, representing the proportion of cells in each cluster in which the gene was detected [a value that already falls in the range (0, 1)]. This process produced two features per TF in each cluster within each dataset (Supplementary Table S8).

Next, a SVM classifier was built by training linear SVM models to classify clusters into their cell classes (CGE/LGE-derived, MGE-derived, and excitatory) using the scaled mean expression and detection rate for each TF in each cluster as input. SVM models were used as implemented in the parsnip R package (v1.1.0, RRID: SCR_024744) using the LiblineaR engine. Performance of the classifier was estimated using four-fold cross-validation, stratifying by class such that each class was balanced across folds.

#### Gene set enrichment analysis of cell type signatures in human tumors

Previously published scRNA-seq data from relevant brain regions and the fetal adrenal glands (Supplementary Table S6) was obtained to derive cell type–specific gene signatures as follows. When available, gene signatures from each cell population were obtained from the original study. Otherwise, gene signatures were derived by computing differentially expressed genes in each cell population compared with all other populations in the same dataset (using the Wilcoxon rank-sum test), sorting by average log_2_ fold change (FC), and filtering out ribosomal genes (defined as having gene symbols containing “RPS,” “RPL,” “MRPS,” and “MRPL”) and mitochondrial genes (defined as having a gene symbol beginning with “MT-”). For signatures with more than 100 genes, the top 100 were used (based on log_2_ FC). Signatures with fewer than 75 genes were excluded from downstream analysis, leaving 390 signatures of similar length for analysis (Supplementary Table S9). To obtain harmonized cell type labels across the dataset, the labels from the original authors were used to manually assign each cluster into one of 21 classes representing broader cell types (Supplementary Table S9).

To identify nondiscriminant signatures, we assembled a background dataset of 210 previously published brain tumors, matched normal tissue, and normal brain samples (nonoverlapping with the tumor samples in this study reported in Supplementary Table S2; ref. 12). The 390 cell type signatures were ranked based on the number of background samples in which they were among the top 10 highest-scoring signatures. A total of 135 signatures appear in the top 10 of any sample, and among these, the top decile (i.e., top 10%, 15 signatures, including 1 tie) were considered to be nondiscriminant and excluded from downstream analyses. Finally, an additional MGE signature (MGE-NR2F1+, MEIS2+ derived from ref. 38; Supplementary Table S9) was excluded as it appeared among the top 10 signatures in 16 of 25 DIPG tumors in this study, incompatible with their site of occurrence in the pons. To confirm the nonspecificity of this signature, we calculated the detection rate of each gene across cell populations of the developing mouse brain (11), which showed that this signature is enriched in all oligodendrocyte precursor (OPC) populations, both in the cortex and the pons, as well as in nonneural cells (endothelial and immune cells). After these filtering steps, the resulting cell type signature panel consisted of a total of 374 discriminant signatures (specified in Supplementary Table S9).

Enrichment of these signatures was then scored in bulk tumor transcriptomes using single-sample gene set enrichment analysis (ssGSEA; ref. 43) adapted from the gene set variation analysis (GSVA) R Bioconductor package (RRID: SCR_021058) with parameters α = 0.75 and norm = FALSE. To visualize the samples in 2D space, the ssGSEA scores were used as input to the *t*-distributed stochastic neighbor embedding dimensionality reduction algorithm (44) implemented in the Rtsne R package (v0.15, RRID: SCR_016342) using perplexity = 10 for intracranial tumors alone and perplexity = 30 for intracranial and extracranial tumors. To test for significantly higher enrichment of signatures in NB-FOXR2 compared with other tumors, *t* tests were performed on the ssGSEA scores in NB-FOXR2 samples versus neuronal tumors [SHH and WNT medulloblastoma and embryonal tumor with multilayered rosettes (ETMR)] or versus glial tumors (DIPGs, IDH-mutant HGGs, and posterior fossa group A ependymoma). *P* values were adjusted using the Benjamini–Hochberg procedure.

#### GSEA of tumor signatures in mouse models

Tumor-specific gene signatures were obtained by pairwise differential expression analyses of bulk human pediatric brain tumors from the following groups: NB-FOXR2 (*n* = 13), DIPG-H3K27M (*n* = 15), MB-WNT (*n* = 10), and ETMR (*n* = 12). For NB-FOXR2 tumors, external samples retrieved from Korshunov and colleagues (3) were excluded from the differential expression to avoid batch effects and to balance the number of samples between the four tumor types. For DIPG-H3K27M, three samples with reported low tumor purity were excluded from the analysis. Differential gene expression analysis was performed using DESeq2 (v1.14.1; ref. 22).

Differentially expressed genes were defined as statistically significant (adjusted *P* value < 0.05) with good expression levels (base mean >100) and large effect size (absolute value of log_2_ FC >1). Genes that were differentially expressed across all comparisons for each tumor type, defined as the top 100 differentially expressed genes by the mean Wald statistic value across comparisons, were used as a gene signature of the tumor type. The R package biomaRt (v2.50.2, RRID: SCR_019214; ref. 45) was used to get mouse gene orthologs for human tumor signatures. Human genes without ortholog, or that did not have a 1-to-1 match between human and mouse, were removed. Ensembl IDs were used to retrieve the mouse and human ortholog pairs and then assigned with the corresponding mouse gene symbol.

Single cells from mouse models were scored for each gene signature using ssGSEA (43) adapted from the GSVA R Bioconductor package (RRID: SCR_021058) with parameters α = 0.75 and norm = FALSE.

### CUT&RUN data analysis

CUT&RUN data were processed using GenPipes (v4.3.2, RRID: SCR_016376; ref. 46) Chromatin Immunoprecipitation Sequencing Pipeline. This includes adapter removal and trimming with Trimmomatic (v0.39; ref. 23), alignment to mm10 with Burrows-Wheeler Aligner (v0.7.17, RRID: SCR_010910; ref. 47), removal of duplicated reads with Picard (v2.26.6, RRID: SCR_006525, https://github.com/broadinstitute/picard), generation of BigWig tracks using Homer (v4.11, RRID: SCR_010881; ref. 48), and peak calling using MACS2 (v2.2.7.1; ref. 49). Peaks called in the FOXR2-p53^LOF^ condition (*n* = 2 replicates) were then filtered to remove false positives, removing peaks that overlapped by at least 10 bp with a peak in one or more of the V5 control replicates (*n* = 2), as well as peaks with −log_10_*q* value equal or lower than 6, with signalValue equal or lower than 6, or with length equal or longer than 10,000 bp. Next, CUT&RUN peaks were intersected with peaks from the Multiome snATAC-seq experiment in the same mouse model. ATAC peaks were called using MACS2 (v2.2.7.1, RRID: SCR_013291; ref. 49) using the CallPeaks function in the Signac (v1.3.0, RRID: SCR_021158; ref. 28) library. Peaks outside of the standard chromosomes (chr 1–19, X, and Y) and peaks in blacklisted regions (retrieved via Signac function blacklist_mm10) were filtered out. Finally, peaks with qValue equal or lower than 4, signalValue equal or lower than 2, and length equal or longer than 10,000 bp were removed. Nucleotide regions that were present in the peaks of the two CUT&RUN replicates as well as in the snATAC-seq were kept as the final set of CUT&RUN peaks.

findMotifsGenome from Homer (v4.11, RRID: SCR_010881; ref. 48) was used to find enriched motifs in filtered CUT&RUN peaks. Input regions were defined as the center of each filtered CUT&RUN peak ± 100 bp. To identify genomic coordinates of specific TF motifs within peaks (e.g., ETS, Sox10), mm10 genomic sequences of filtered CUT&RUN peaks were identified with bedtools (v2.30.0; ref. 25) and provided as input to function matchMotifs from R package motifmatchr (v1.20.0, https://github.com/GreenleafLab/motifmatchr).

### Ethics approval

All work was performed in accordance with the Declaration of Helsinki. This study was approved by the Institutional Review Board of the respective institutions from which the samples were collected. Written informed consent was obtained from patients and/or guardians through protocols approved by the Institutional Review Board at each institution. Protocols for this study involving collection of patient samples and information were approved by the Research Ethics and Review Board of McGill University and Affiliated Hospitals Research Institutes and the Research Ethics Board at the Hospital for Sick Children. Protocols involving IUE and engraftment for mouse models were approved by the University of Cambridge AWERB and carried out under a UK Home Office License (PPL PP2303899) in accordance with the Animals (Scientific Procedures) Act 1986.

### Data availability

Raw data for human tumors (bulk RNA-seq, snRNA-seq, and scMultiome sequencing) have been deposited in the European Genome-phenome Archive under accession number EGAS00001007247. Raw and processed data for murine models (bulk RNA-seq, scMultiome, and CUT&RUN) have been deposited to Gene Expression Omnibus under accession number GSE270666. Processed data, including bulk RNA-seq (counts and differential expression analyses), BigWig files, and labeled single-cell expression matrices have been deposited to Zenodo at https://doi.org/10.5281/zenodo.13750919. Data from the Gartlgruber 2021 (21) dataset of EC-NB (bulk RNA-seq) analyzed in this study were obtained as count matrices from a public web app provided by the authors (https://nbseb087.dkfz.de/project_NB_SE_viz/). Data from the TARGET dataset of EC-NB (bulk RNA-seq) analyzed in this study were obtained as count matrices from the public Genomic Data Commons cancer portal (https://portal.gdc.cancer.gov/). Citations and sources for previously published datasets of tumors, normal brain, and adrenal gland used in this study are provided in Supplementary Tables S2, S6, and S7. Informed consent did not extend to sharing of raw data for one of the patients included in this study, and therefore only processed data are available for this patient. All other raw data generated in this study are available upon request from the corresponding author.

The code to reproduce the main results included in the paper is available at https://github.com/fungenomics/NB-FOXR2 and archived on Zenodo at https://www.doi.org/10.5281/zenodo.13755695.

## Results

**Figure 1.**
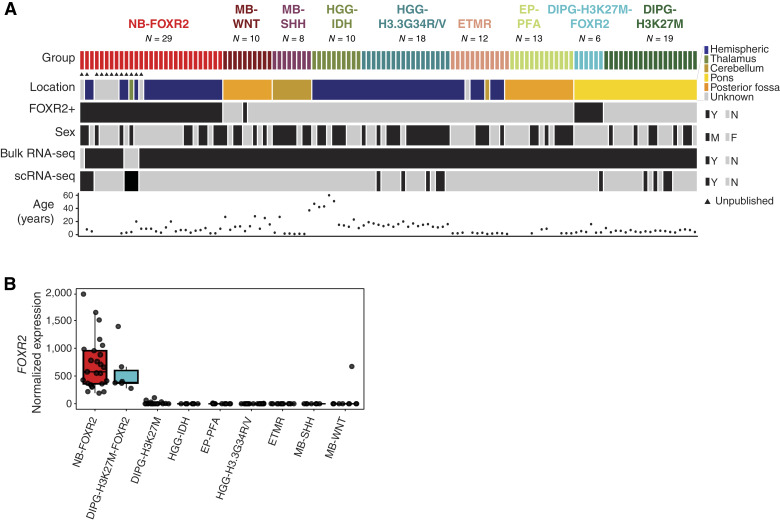
A cohort of CNS neuroblastoma with *FOXR2* activation (NB-FOXR2). **A,** Oncoprint of assembled cohort of NB-FOXR2 and other brain tumor entities, profiled by bulk and/or scRNA-seq. **B,** Expression of *FOXR2* by bulk RNA-seq across brain tumor subtypes. DIPG-H3K27M-FOXR2, DIPG, H3K27M altered, and *FOXR2* activated; DIPG-H3K27M, DIPG, H3K27M altered; EP-PFA, posterior fossa group A ependymoma; ETMR, embryonal tumors with multilayered rosettes; HGG-IDH, HGG, IDH-mutant; HGG-H3.3G34R/V, HGG, H3.3G34R/V altered; MB-SHH, SHH medulloblastoma; MB-WNT, WNT medulloblastoma.

To define the transcriptional landscape of NB-FOXR2, we assembled a cohort of 29 NB-FOXR2 patient tumor samples profiled at the bulk (*N* = 25) and single-cell levels (*N* = 6; Fig. 1; Supplementary Fig. S1; Supplementary Table S2). Given the neuronal and oligodendroglial features of these tumors, we included other pediatric brain tumor entities, consisting of both glial (ependymomas and HGGs) and neuronal (ETMR and medulloblastoma) tumor types (*N* = 96; Supplementary Tables S2–S4). Finally, to determine whether transcriptional characteristics of NB-FOXR2 are a downstream effect of *FOXR2* expression, we also included bulk RNA-seq for *FOXR2*+ gliomas and a large collection of childhood EC-NBs (*N* = 707 from ref. 21 and TARGET).

### TF fingerprints define telencephalon progenitor domains

As a first step to map NB-FOXR2 to specific neural lineages in the normal brain, we derived a panel of TFs that are sufficient to infer the progenitor domain of origin for cortical neurons with high sensitivity in the postnatal human brain. In other words, we sought combinations of TFs specified during fetal development, maintained during neuronal differentiation, and persistent during postnatal development to adulthood.

**Figure 2.**
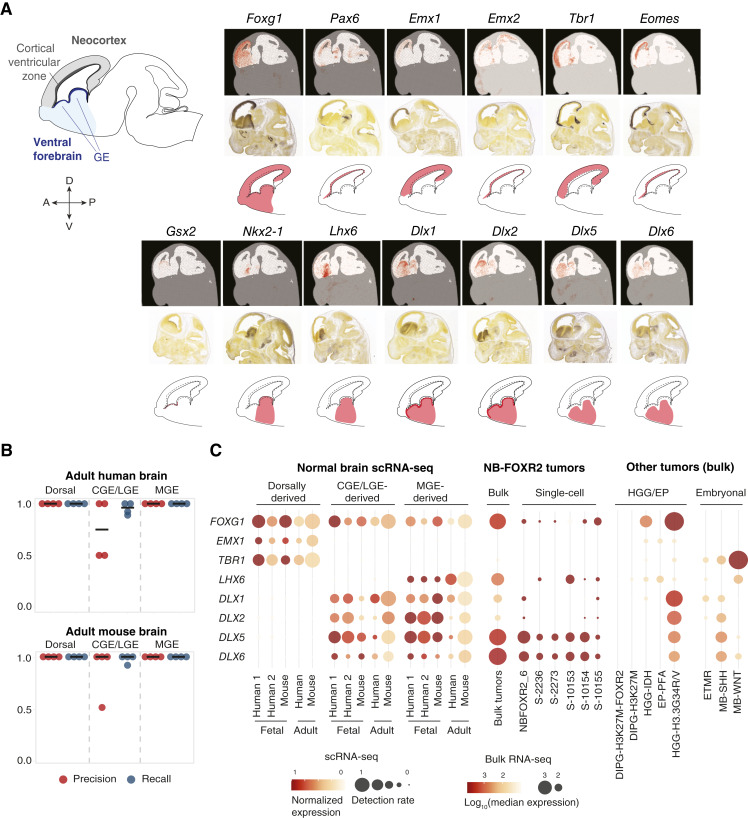
NB-FOXR2 expresses a TF fingerprint of the MGE. **A,** Expression of telencephalon-patterning TFs in the embryonal mouse brain. Top, stereo-seq *in situ* RNA expression of telencephalon-patterning TFs in E14 mouse (Chen and colleagues, ref. 75; https://db.cngb.org/stomics/mosta/spatial/). Middle, RNA *in situ* hybridization in E13 mouse (Allen Brain Atlas, https://developingmouse.brain-map.org/). Bottom, schematic of the mouse brain (sagittal section) at E13, and schematized expression of each TF in the telencephalon. **B,** Evaluation of SVM-based classification of neuron clusters from normal fetal and adult brain scRNA-seq datasets based on expression levels of telencephalon-patterning TFs in each cluster. Precision and recall metrics are calculated across clusters in each class in each dataset and are shown for each fold of a four-fold cross-validation experiment (one point per fold) for the adult datasets. Top, adult human dataset; bottom, adult mouse dataset. **C,** Expression of telencephalon TF fingerprint across normal brain and tumor datasets. Left, expression in clusters of single cells from reference datasets of the human and mouse fetal and adult brains aggregated by class (dorsal, CGE/LGE, and MGE). Scaling was performed within each dataset. Human 1, data from Yu and colleagues (38). Human 2, data from Shi and colleagues (39). Middle, expression in NB-FOXR2 tumors profiled by bulk RNA-seq and in malignant cells of NB-FOXR2 tumors profiled by scRNA-seq. For single-cell tumors, scaling was performed between malignant and normal cells of the same sample (normal cells not shown). For bulk RNA-seq, bubble size encodes log_10_-transformed median normalized expression across samples in each brain tumor subtype. Right, expression in other brain tumors profiled by bulk RNA-seq. DIPG-H3K27M-FOXR2, DIPG, H3K27M altered, and *FOXR2* activated; DIPG-H3K27M, DIPG, H3K27M altered; EP-PFA, posterior fossa group A ependymoma; ETMR, embryonal tumors with multilayered rosettes; HGG-IDH, HGG, IDH-mutant; HGG-H3.3G34R/V, HGG, H3.3G34R/V altered; MB-SHH, SHH medulloblastoma; MB-WNT, WNT medulloblastoma.

For this, because most NB-FOXR2 tumors present in telencephalon-derived brain regions (in the cortical lobes and in the basal ganglia), we established a gene panel of discriminant patterning genes for the telencephalon (Fig. 2A). This included *FOXG1*, a pan-telencephalon marker (50, 51); *PAX6*, *EMX1*, and *EMX2*, markers of the neocortex; and *TBR1* and *EOMES*, marking intermediate precursors of excitatory neurons, which are dorsally derived (52). To account for ventral progenitor domains, we included *GSX2* (53) and *NKX2-1* (54), which mark the LGE and MGE, respectively; *LHX6*, which is expressed downstream of *NKX2-1* (55); and *DLX1/2/5/6*, which are critical for differentiation of all ventral telencephalon–derived GABAergic neurons (56).

Next, as NB-FOXR2 tumors are diagnosed in childhood, we assessed whether these TFs are sufficient to infer the progenitor domain of origin for cortical neurons with high sensitivity in the postnatal human brain. For this, we used five annotated scRNA-seq datasets for the developing and adult brain, comprising 258,532 cells and 524 cell populations (Supplementary Table S6). These studies, focused on telencephalon prenatally and cortex postnatally, profiled the human 8 to 12 PCW fetal subpallium (38, 39) and the adult temporal cortex (41). In mouse, in turn, they cover the E10-P6 telencephalon (11, 12) and the adult isocortex (40). For each dataset, we used the cell populations as defined in the original studies and retained only neuronal lineage populations that were unambiguously MGE-derived, LGE/CGE-derived, or excitatory (dorsally derived). Next, we trained linear SVMs to classify cell populations from these three classes into their progenitor domain of origin based on two features per gene in this panel: the proportion of cells in the population in which each TF is detected (detection rate) and the average expression of each TF (Supplementary Table S8). In a cross-validation experiment, this classifier exhibited a median sensitivity (recall) across folds ranging from 96% to 100% for the postnatal brain (Fig. 2B). Thus, this small panel of telencephalon patterning TFs is highly predictive of anatomical origins of cortical neurons even in the adult brain.

Finally, we identified the subset of TFs within this panel that persist during postnatal development to adulthood (Fig. 2C; Supplementary Fig. S2). The combination of *FOXG1*, *LHX6*, and *DLX1/2/5/6* marked the MGE during fetal development, and in adulthood, was exclusively expressed in MGE-derived inhibitory neurons (Fig. 2C; Supplementary Fig. S2). The combination of *FOXG1*, *EMX1*, and *TBR1* was exclusive to excitatory neurons. Thus, in mature or differentiated cortical neurons, specific combinations of TFs act as fingerprints of the progenitor domain in which they were born. The maintenance of these TFs in specific neuronal lineages from embryonic development to adulthood suggests that their combinatorial expression is tightly regulated and unlikely to be acquired by chance, for example, during oncogenic transformation.

### NB-FOXR2 express a MGE TF fingerprint

We next assessed expression of the telencephalon TF fingerprints in brain tumor samples. NB-FOXR2 tumors matched the MGE fingerprint (Fig. 2C), expressing *FOXG1*, *NKX2*-*1*, *LHX6*, and *DLX5/6* and lacking the LGE marker *GSX2*, which was active instead in H3.3G34R/V gliomas, as expected (Supplementary Fig. S3A; ref. 10). They also lacked expression of all dorsal markers (*PAX6*, *EMX1*, *EMX2*, *TBR1*, and *EOMES*). No other brain tumor type in our study matched this fingerprint (Supplementary Fig. S3A). This result, consistent at the bulk and single-cell levels, suggests that NB-FOXR2 tumors maintain a coherent regional identity of the ventral telencephalon and specifically the MGE.

**Figure 3.**
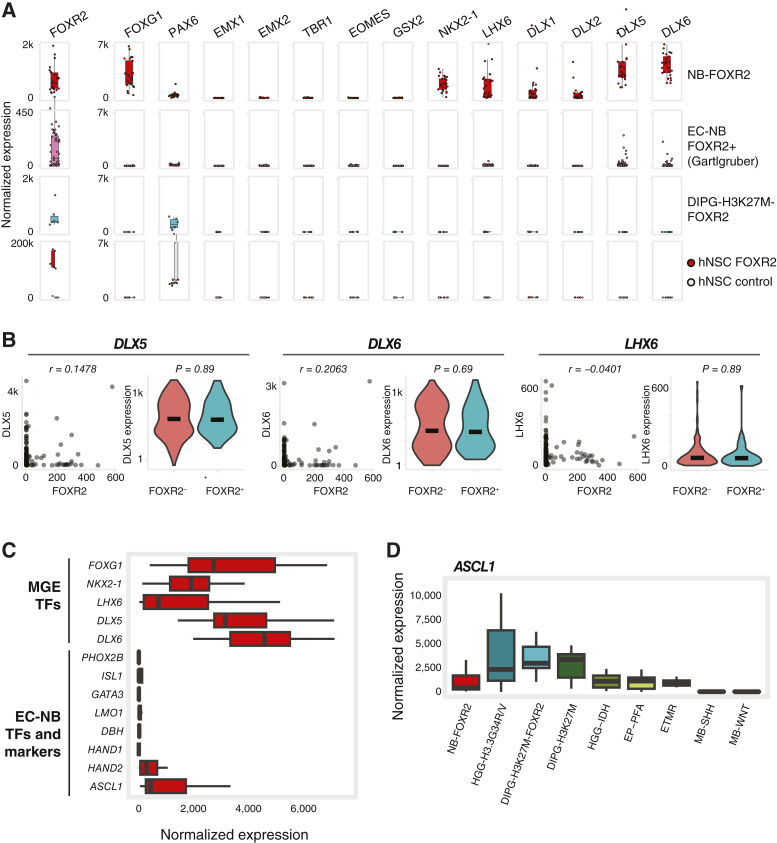
*FOXR2* does not induce expression of MGE or EC-NB TFs. **A,** Bulk RNA-seq expression of telencephalon patterning TFs. Top and third rows, tumor samples from this study cohort. Second row, EC-NB samples (Gartlgruber and colleagues, ref. 21) subset to high risk, stage 4 samples with *FOXR2* expression (normalized expression >2). Bottom row, H9 human NSCs transduced with HA-FOXR2 or controls transduced with HcRed (Tsai and colleagues, ref. 7). All expression box plots except *FOXR2* have a *y*-axis maximum value of 7,000. **B,** Comparison of TF expression with *FOXR2* status in EC-NB (Gartlgruber and colleagues, ref. 21) subset to high risk, stage 4 samples (*n* = 229). Left, correspondence between *FOXR2* expression and TF expression, annotated with Pearson correlation. Right, TF expression in samples split by *FOXR2* positivity, annotated with *P* value (Wilcoxon test). *FOXR2*+ tumors, normalized expression >2. *Y*-axis for *DLX5* and *DLX6* violin plots are log_10_-scaled. **C,** Bulk RNA-seq expression in NB-FOXR2 of MGE and EC-NB TFs and markers. **D,** Bulk RNA-seq expression of *ASCL1* across pediatric brain tumor types. DIPG-H3K27M-FOXR2, DIPG, H3K27M altered, and *FOXR2* activated; DIPG-H3K27M, DIPG, H3K27M altered; EP-PFA, posterior fossa group A ependymoma; ETMR, embryonal tumors with multilayered rosettes; HGG-IDH, HGG, IDH-mutant; HGG-H3.3G34R/V, HGG, H3.3G34R/V-altered; MB-SHH, SHH medulloblastoma; MB-WNT, WNT medulloblastoma.

Expression of the MGE TF fingerprint in NB-FOXR2 tumors could be a downstream effect of FOXR2 activity. To decouple the oncogenic effect of FOXR2 from the lineage of origin, we analyzed patient samples expressing FOXR2 in different tumor contexts (EC-NBs and DIPGs) and *FOXR2*-transduced human neural stem cell lines (Fig. 3A; ref. 7). *FOXR2*+ DIPGs lacked expression of all telencephalon-patterning TFs except for *PAX6*. Similarly, EC-NB also lacked overall expression of these TFs, a result replicated across datasets (Supplementary Fig. S3B) and consistent with their distinct origins in neural crest–derived lineages. A few EC-NB samples, however, showed expression of *DLX5/6* or *LHX6*. We thus analyzed the coexpression of these genes with *FOXR2* and found that they are completely uncoupled in both EC-NB cohorts (Fig. 3B; Supplementary Fig. S3C). Conversely, NB-FOXR2 lacked expression of the characteristic TFs and marker genes of EC-NB (Fig. 3C), except for *ASCL1*, known for its essential role in multiple CNS neural lineages (57) and expressed across brain tumor types (Fig. 3D), and, to a lower level, *HAND2*. Finally, in neural stem cells, although both *FOXR2*+ and control cells expressed *PAX6*, neither condition expressed any other telencephalon-patterning TFs.

Altogether, our data indicate that NB-FOXR2 tumors express a TF fingerprint unique to MGE-derived neuron lineages, absent in other pediatric brain tumors or in EC-NBs. The absence of this fingerprint in *FOXR2*+ DIPGs, *FOXR2*+ EC-NB, and a cell line transduced with *FOXR2* suggests that this fingerprint is not likely to be a consequence of *FOXR2* activation and instead may be maintained from the tumor lineage of origin.

### NB-FOXR2 tumors map to MGE-derived interneurons with OPC features

**Figure 4.**
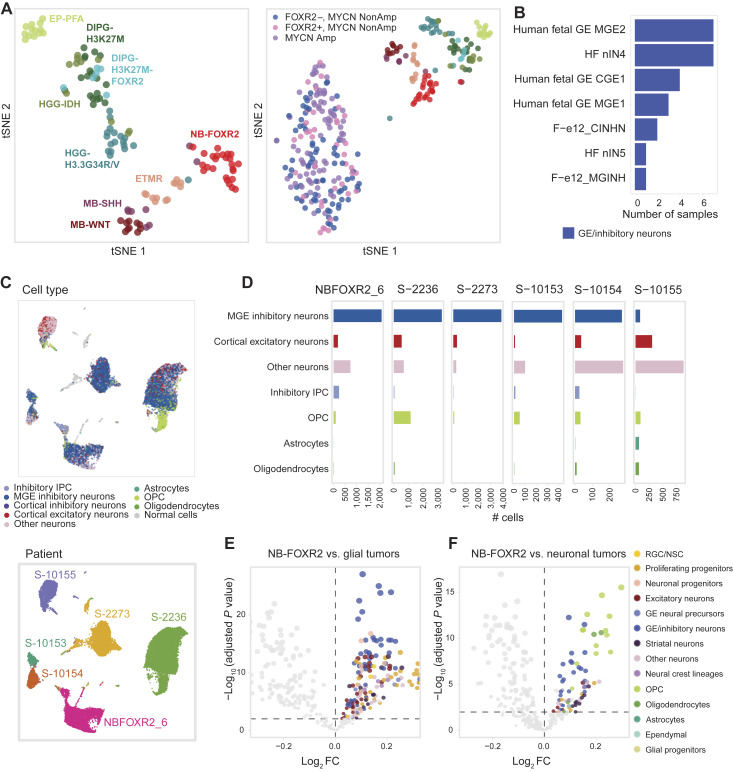
NB-FOXR2 tumors transcriptionally resemble interneurons and OPC cells. **A,***t*-Distributed stochastic neighbor embedding (*t*-SNE) of bulk RNA-seq profiles of pediatric brain tumors (left) and pediatric brain tumors with stage 4 high-risk EC-NBs from Gartlgruber and colleagues (ref. 21; right) using ssGSEA scores for *N* = 374 cell type–specific gene signatures from reference scRNA-seq datasets. *t*-Distributed stochastic neighbor embedding perplexity = 10 for left and perplexity = 30 for right. Color legend for pediatric brain tumors in the right panel matches labels in the left panel. *FOXR2*+ tumors, normalized expression >2. **B,** Tally of the top-scoring signatures across NB-FOXR2 bulk tumors (*N* = 25). The *X*-axis indicates the number of samples in which each signature is the top match by ssGSEA (Supplementary Table S10). HF nIN4/HF nIN5, human fetal interneuron 4 and 5; F-e12 CINHN, forebrain E12 cortical inhibitory neurons; F-e12 MGINH, forebrain E12 MGE inhibitory neurons; human fetal GE MGE/CGE, human fetal GE progenitors. **C,** Uniform Manifold Approximation and Projection joint representation of NB-FOXR2 tumors (*N* = 6) profiled by snRNA-seq. Top, points colored by consensus cell type annotation based on a reference dataset of the developing mouse brain. Gray, nonmalignant cells. Bottom, points colored by sample. Samples are joined without integration or batch correction. **D,** Number of malignant cells per consensus projected cell type across NB-FOXR2 tumors profiled by snRNA-seq (*N* = 6). Cell classes comprising >2% cells per sample are shown. **E,** Volcano plot for differential ssGSEA enrichment of cell type–specific signatures in bulk NB-FOXR2 compared with other tumor entities. Point size reflects −log_10_(adjusted *P* value). Differential testing was performed with *t* tests between NB-FOXR2 tumors and glial tumors (HGGs, DIPGs, posterior fossa group A ependymoma), followed by multiple testing correction using the Benjamini–Hochberg procedure. A positive FC represents enrichment in NB-FOXR2. Each point represents one signature, and signatures with positive log FCs and adjusted *P* value < 0.01 are colored. Color legend as in **F**. **F,** Differential ssGSEA enrichment as in **E**, comparing NB-FOXR2 tumors with neuronal tumors (ETMR, MB-SHH, and MB-WNT). Amp, amplified; EP-PFA, posterior fossa group A ependymoma; IPC, intermediate progenitor cell; NonAmp, nonamplified; RGC, radial glial cells.

To systematically map tumors to the developing brain, we next compiled a comprehensive single-cell resolution reference of the prenatal telencephalon and the postnatal ventricular/subventricular zone from eight scRNA-seq studies (Supplementary Table S6). We also included three references for the fetal adrenal gland, containing lineages relevant for EC-NB (Supplementary Table S6; refs. 58, 59). Using this reference and a background dataset of 210 independent brain tumors and normal brain samples (11, 12), we derived 374 discriminant cell type gene signatures spanning all major cell classes (Supplementary Table S9). ssGSEA (43) scores for these signatures are sufficient to segregate bulk tumors by type (Fig. 4A). Strikingly, in all cases, GE-derived cell types were the top-scoring signature for NB-FOXR2 tumors (Fig. 4B; Supplementary Fig. S4A and S4B; Supplementary Table S10). In contrast, a human fetal adrenal gland sympathoblastic signature was the highest scoring in 552/579 (95%) of EC-NBs, consistent with their hypothesized cell of origin (58, 59), and served as a positive control for this analysis. Similarly, in other pediatric tumor types, top-scoring signatures also reflected their postulated lineages of origin (Supplementary Fig. S4C).

To map single-cell tumor profiles to the normal brain, in turn, we trained machine learning–based cell annotation tools on a telencephalon reference dataset (11) to define a consensus label for each cell (Fig. 4C and D). Four of six tumors displayed minor populations of OPC-like cells (Fig. 4D), consistent with the known expression of oligodendroglial markers in these tumors, including *OLIG2* and *SOX10* (Supplementary Fig. S5A). Importantly, in all but one tumor, a large proportion (39%–85%) of malignant cells with consensus labels was predicted to be MGE-derived inhibitory neurons (Fig. 4D). Furthermore, analysis of canonical neuronal markers (Supplementary Fig. S5B) showed that malignant cells expressed, in addition to pan-neuronal markers (*SNAP25* and *STMN2*), pan-GABAergic markers (*GAD1* or *GAD2*) or other interneuron markers (*LHX6*, *SST*, *DLX5*, *DLX6*, or *NXPH1*) with negligible expression of the pan-glutamatergic marker *SLC17A7* (Supplementary Fig. S5C), including in the tumor in which automated labeling had failed to identify inhibitory neuronal populations.

Finally, we compared enrichment scores of cell type signatures among tumor groups. Compared with glial brain tumors, NB-FOXR2 were enriched for various neuronal and neuronal progenitor signatures, but the most significantly enriched signatures were, once again, for cortical interneurons (Fig. 4E). Compared with neuronal brain tumors, in turn, NB-FOXR2 tumors were significantly enriched for signatures of OPCs, cortical interneurons, and striatal neurons (Fig. 4F). Altogether, based on complementary approaches for mapping tumors to their normal brain counterpart, using GSEA and machine learning–based single-cell projections, we find that NB-FOXR2 tumors resemble MGE-derived cortical interneurons, with concurrent OPC-like features.

### *Foxr2* is oncogenic in the ventral telencephalon *in vivo*

**Figure 5.**
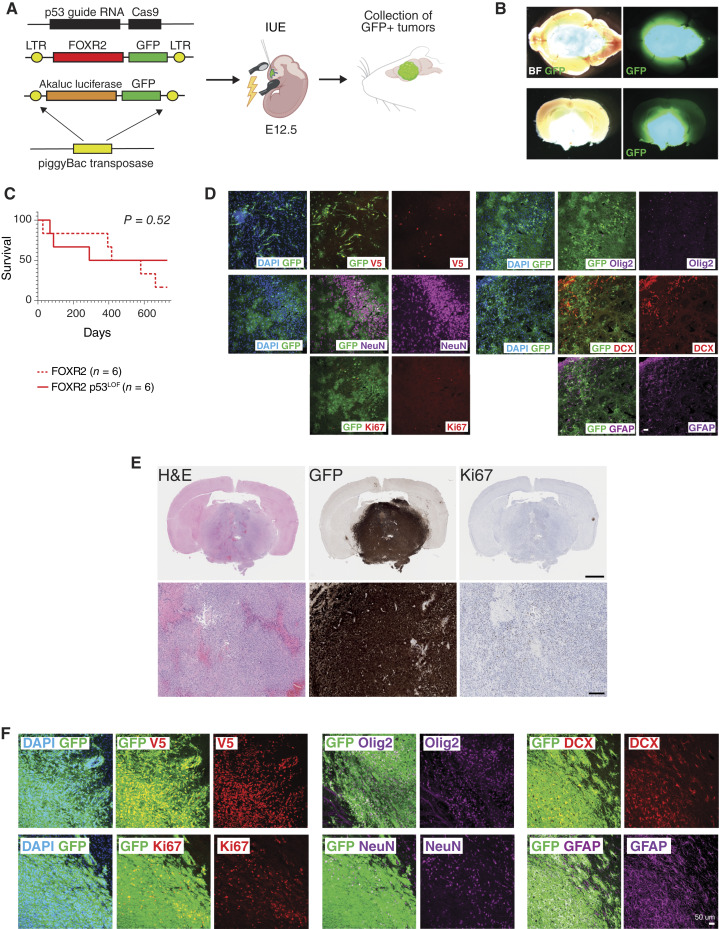
*Foxr2* is oncogenic in the ventral telencephalon in mice. **A,** Schematic describing the IUE-based strategy to model *FOXR2*-driven brain tumors. PiggyBac and CRISPR vectors are delivered into neural stem cells in the GEs at E12.5. After birth, mice develop tumors and are euthanized when neurologic symptoms become apparent. **B,** Representative brightfield (BF) and fluorescence (GFP) images of a GFP+ *Foxr2*-driven tumor (top, whole brain; bottom, coronal section). **C,** Kaplan–Meier survival curves of tumor-bearing mice carrying *Foxr2* overexpression alone (*Foxr2* alone, *n* = 6) or in combination with p53 LOF (*Foxr2* p53^LOF^, *n* = 6). Statistical comparisons using the log-rank Mantel–Cox test are described in Supplementary Table S1. **D,** Immunofluorescence of *Foxr2* alone tissue in coronal sections from the striatum. Lesions driven by *Foxr2* overexpression alone are GFP+ and colocalize with V5. Cells harboring *Foxr2* overexpression alone possess low levels of Ki67 and Olig2. However, cells in these lesions are positive for NeuN, DCX, and GFAP. Scale bar, 50 μm. **E,** IHC detection of hematoxylin and eosin (H&E), GFP, and Ki67 in coronal forebrain sections from *Foxr2* p53^LOF^ tumor-bearing symptomatic mice. Tumor cells are GFP+. Scale bars, 1 mm in the low-magnification panels and 100 μm in the high-magnification panels. **F, **Immunofluorescence for GFP, Foxr2-V5, Ki67, Olig2, NeuN, DCX, and GFAP in coronal forebrain sections from *Foxr2* p53^LOF ^tumor-bearing symptomatic mice. Cells within the tumor in the striatum are GFP+, colocalize with V5, and many are actively proliferating, as indicated by the presence of Ki67. Individual tumor cells are also positive for Olig2, NeuN, DCX, and GFAP. Scale bar, 50 μm.

To evaluate an oncogenic role for *Foxr2* overexpression and the contribution of the GE niche *in vivo*, we developed mouse models using an IUE-based approach (Fig. 5A). As NB-FOXR2 patients carry chromosome 1q gain (1), recently shown to phenocopy p53 loss-of-function (p53^LOF^) via *MDM4* overexpression (Supplementary Fig. S1C–S1E; ref. 6), we developed models carrying *Foxr2* either alone or in combination with p53^LOF^. This IUE method delivers genetic alterations into discrete neural stem cell niches in the embryo at precise developmental timepoints, allowing spatiotemporal control of tumor development in specific brain locations. piggyBac transposon and CRISPR vectors were used to introduce *Foxr2* overexpression alone or in combination with p53^LOF^ in the GE at embryonic day 12.5 (E12.5). Transient expression of piggyBac transposase and Cas9 ensured permanent recombination in successfully targeted progenitor cells. To enable bioluminescence imaging *in vivo*, a vector expressing Akaluc luciferase was codelivered. The vectors carrying *Foxr2* and Akaluc expressed GFP downstream from a modified 2A peptide (PQR), allowing immunochemical detection of tumor cells with GFP (Fig. 5B). The *Foxr2* vector was also C-terminally tagged with a V5 label.

Models overexpressing *Foxr2* alone developed neurologic symptoms with high penetrance (83%, Fig. 5C; Supplementary Table S1), including lethal seizures and epilepsy. They harbored nonproliferative lesions in the cortex or the striatum, brain regions normally containing GE-derived cells. Neurons and glia expressing *Foxr2* were found to be mislocalised, forming inappropriate heterotopic clusters (Fig. 5D). They displayed low levels of Ki67, Olig2, and DCX and higher levels of the mature neuronal marker NeuN, suggesting that overexpression of *Foxr2* alone may lead to lesions that harbor differentiated cells and fewer proliferative or stem-like tumor cells (Fig. 5D).

In contrast, *Foxr2* p53^LOF^ led to high-grade aggressive tumors with 50% penetrance (Fig. 5C; Supplementary Table S1), appearing as large GFP^+^ lesions in the striata of symptomatic mice, displaying infiltrative margins, and containing pleiomorphic and occasionally multinucleated tumor cells (Fig. 5E). Ki67 labeling indicated a high mitotic index, with 15% of cells actively proliferating (Fig. 5E). As compared with the *Foxr2* alone model, *Foxr2* p53^LOF^ tumors expressed higher levels of the oligodendroglial marker Olig2 and the neuronal progenitor marker DCX, and retained intermittent expression of the mature neuronal marker NeuN (Fig. 5F), recapitulating the mixed neuronal/oligodendroglial profile of human NB-FOXR2.

**Figure 6.**
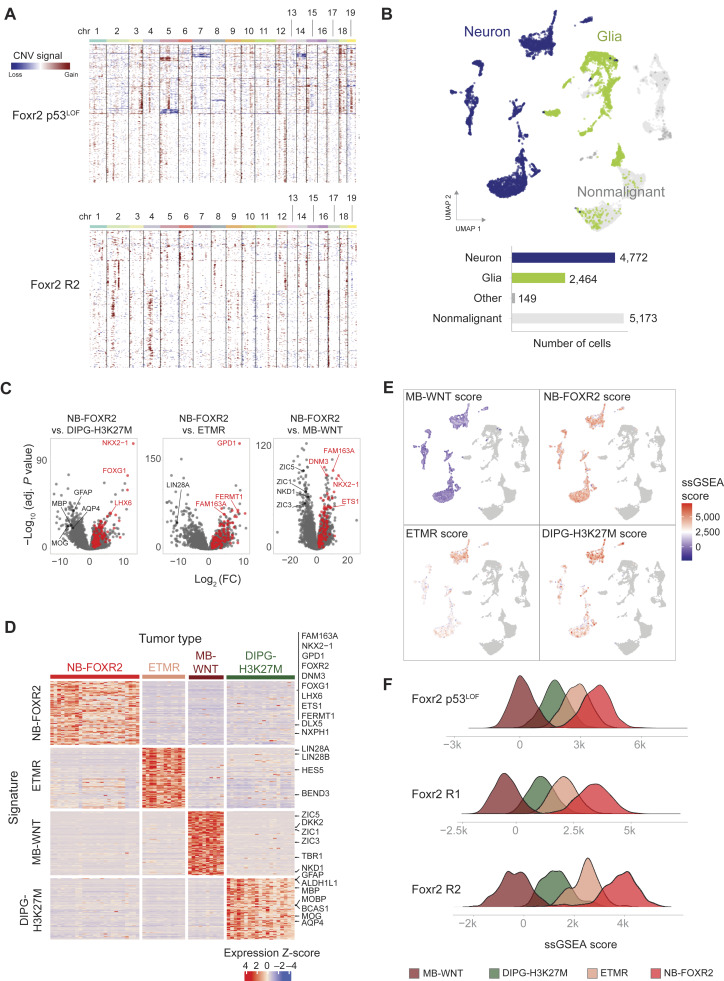
Mouse models transcriptomically recapitulate human NB-FOXR2. **A,** Heatmap showing CNVs in *Foxr2* p53^LOF^ and *Foxr2* alone models, computed with inferCNV. **B,** Top, Uniform Manifold Approximation and Projection (UMAP) joint representation of mouse model single-cell datasets (*n* = 3) without integration or batch correction. Cells colored by broad cell class derived from automated cell type annotation, with nonmalignant cells colored in gray. Bottom, bar plot quantification of number of cells per group in UMAP. **C,** Volcano plots of differentially expressed genes between bulk RNA-seq tumors in NB-FOXR2 compared with MB-WNT, DIPG-H3K27M, and embryonal tumors with multilayered rosettes (ETMR) groups. Points colored in red are the top 100 differentially expressed genes in common across comparisons, used as a tumor signature of NB-FOXR2. **D,** Validation of tumor signature expression specificity in each tumor type. Heatmap showing bulk RNA expression of tumor signature genes (rows) in each tumor sample (columns). Row values are *Z*-scored. **E,** UMAP joint representation of mouse models as in **B**, with malignant neuron-like cells colored by the ssGSEA score of each tumor signature. **F,** Distribution of tumor signature ssGSEA scores in malignant neuron-like cells of murine models. Each row is one sample. Adj, adjusted; chr, chromosome; DIPG-H3K27M, DIPG, H3K27M altered; MB-WNT, WNT medulloblastoma.

To further characterize the *Foxr2*-induced models, we profiled RNA and chromatin accessibility at single-cell resolution using the 10x Multiome technology (*Foxr2* p53^LOF^, *n* = 1; *Foxr2*, *n* = 2). In total, we obtained 14,125 cells passing QC (Supplementary Table S5), which we annotated using a consensus of machine-learning classifiers trained on murine forebrain references (Supplementary Table S6). Inference of copy-number alterations revealed that all models displayed some degree of chromosomal abnormalities (Fig. 6A; Supplementary Fig. S6A and S6B), including the ones induced by *Foxr2* alone, despite their lack of bona-fide proliferating tumor lesions. All models also recapitulated the dual neuronal/glial features of NB-FOXR2 tumors (Fig. 6B), with a large proportion (49%) of the neuron-like cells mapping to a mix of GE-derived neuronal types (Supplementary Fig. S6C) and with malignant cells expressing canonical neuronal and glial markers (Supplementary Fig. S6D).

To assess the similarity of murine models to the human disease, we derived tumor-specific gene signatures from human RNA-seq samples. For this, we performed pairwise differential gene expression analysis (Fig. 6C) and identified the top 100 genes that were specific to each tumor type across all comparisons (Fig. 6D). Enrichment analysis of these signatures in each individual murine cell by ssGSEA showed that, in all models, neuron-like malignant cells scored higher for NB-FOXR2 signatures than for any other tumor type (Fig. 6E and F), indicating that these cells are transcriptionally closer to human NB-FOXR2 than to other pediatric brain tumors. In turn, glial-like malignant cells showed more variable patterns of enrichment, with higher scores for signatures from DIPG-H3K27M than from other tumors, as expected given the glial nature of DIPGs (Supplementary Fig. S6E).

**Figure 7.**
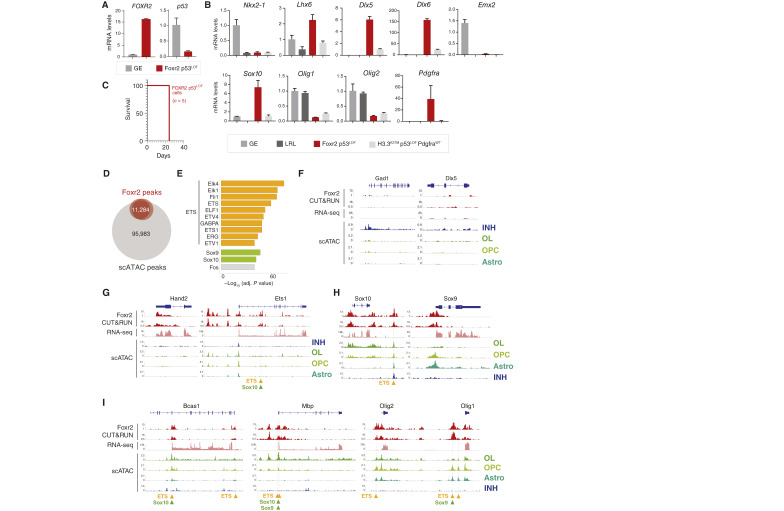
Foxr2 chromatin binding sites in *Foxr2* p53^LOF^ neurosphere show enrichment in ETS and glial pathways. **A,** Validation of FOXR2 overexpression and p53 downregulation in *ex vivoFoxr2* p53^LOF^ neurospheres by qRT-PCR. **B,** Validation of *Nkx2-1*, *Lhx6*, *Dlx5*, *Dlx6*, *Emx2*, *Sox10*, *Olig1*, *Olig2*, and *Pdgfra* expression in *Foxr2* p53^LOF^ neurospheres by qRT-PCR. *Foxr2* p53^LOF^ cell lines are positive for MGE markers and mirror the transcriptomic profile observed in patients. **C,** Kaplan–Meier curves depicting survival following orthotopic (striatal) injection of 150,000 *Foxr2* p53^LOF^ cells (*n* = 5). **D,** Intersection of Foxr2 CUT&RUN peaks (*n* = 2 replicates) with ATAC peaks from single-cell multiome profiling of the murine *Foxr2* p53^LOF^ model, used for filtering peaks. **E,** Top enriched TF motifs in filtered Foxr2 CUT&RUN peaks by adjusted *P* value. For visualization, the figure includes the top 13 motifs, colored and grouped by TF types. **F,** Tracks at *Gad1* and *Dlx5* genomic regions showing Foxr2 CUT&RUN and bulk RNA-seq in *Foxr2* p53^LOF^ murine cell lines and pseudobulk ATAC tracks of malignant cells by cell type from single-cell multiome profiling of *Foxr2* p53^LOF^ mouse model tissue. **G,** Tracks as in **F** at genomic regions for *Hand2* and *Ets1*. **H,** Tracks as in **F** at genomic regions for Sox family glial TFs. **I,** Tracks as in **F** at genomic regions for key oligodendrocyte lineage genes. Adj, adjusted; Astro, astrocytes; INH, inhibitory neurons; LRL, lower rhombic lip; OL, oligodendrocytes.

We next derived two cell lines from *Foxr2* p53^LOF^ tumors and grew them in serum-free media as neurospheres. We validated *Foxr2* overexpression and p53 downregulation, demonstrating that these cell lines retain the alterations introduced in the embryos (Fig. 7A). These *ex vivo* cell lines recapitulated expression patterns present in human NB-FOXR2 tumors, displaying high levels of the GE-specific TFs *Lhx6*, *Nkx2-1*, *Dlx5*, and *Dlx6*, which suggest that they arose from a medial GE progenitor lineage (Fig. 7B). They are also positive for the oligodendroglial markers *Sox10* and *Olig2*, mirroring the mixed neuronal and oligodendroglial transcriptomic profiles of patient tumors (Fig. 7B). We evaluated the engraftment potential of these neurosphere lines in syngeneic, immunocompetent C57BL/6J mice. *Foxr2* p53^LOF^ cells were orthotopically engrafted into the striatum, in which they produced 100% penetrant tumors with a very short latency of just 24 days (Fig. 7C; Supplementary Table S1). Engrafted tumors were positive for GFP and V5 and expressed high levels of Ki67, Olig2, and DCX, recapitulating the *de novo* model in this aspect (Supplementary Fig. S7). Altogether, our data show that *Foxr2 *p53^LOF^ delivered directly to the embryonic GE niche has oncogenic potential and that the resulting tumors recapitulate molecular features that are specific to human NB-FOXR2.

### Foxr2 co-opts ETS and glial pathways in GE-derived lineages

To map FOXR2 transcriptional targets, uncoupling them from lineage-of-origin programs, and to dissect potential oncogenic mechanisms, we profiled gene expression by RNA-seq and Foxr2 DNA-binding sites by CUT&RUN in a *Foxr2* p53^LOF^ line. We identified 11,284 binding sites reproducible across replicates (*n* = 2). Of these sites, 96.5% overlapped regions of open chromatin in the mouse tumor single-cell data, indicating that the overall chromatin landscape of the *in vivo* model was well recapitulated in the neurosphere line (Fig. 7D).

Motif enrichment analysis of Foxr2binding sites showed a clear association with the ETS family of TFs, which comprised 11 of the top 15 most significantly enriched motifs (Fig. 7E), consistent with earlier findings that Foxr2 activates ETS transcriptional programs (7). We next inspected genomic regions surrounding patterning and lineage TFs, as well as NB-FOXR2–specific genes. Foxr2 binding was not detected at GE-patterning TFs or interneuron genes (Fig. 7F), suggesting once again that expression of these genes reflects their likely cellular origins. However, Foxr2 binding sites were present in promoters of ETS TF genes, such as *Ets1* (Fig. 7G); at genes from the NB-FOXR2 signature, such as *Gpd1*, *Dnm3*, and *Nxph1*; and at *Hand2*, a gene also upregulated in EC-NB (Figs. 3C and 7G).

Among the top enriched motifs were also Sox9, which coordinates the initiation of gliogenesis (60–62), and Sox10, essential for terminal oligodendrocyte differentiation (60, 63). In contrast to the lack of binding around GE and interneuron genes, we observed strong signal for Foxr2 binding on promoters and regulatory elements of *Sox9* and *Sox10* in regions of open chromatin specific to glial-like tumor cells, correlating with expression of those genes in the RNA-seq data (Fig. 7H). Previous work has shown that expressions of Sox9 with Nfia and Nfib (61, 62) and Sox10 with Olig2 and Zfp536 (63) are sufficient for direct lineage conversion to glia. We found the same pattern of Foxr2 binding and RNA expression in all these genes, as well as in downstream canonical oligodendrocyte markers (Fig. 7I), suggesting that oligodendroglial reprogramming could be a downstream effect of *Foxr2* expression in a GE-derived cellular background and potentially explaining the mixed neuronal/oligodendroglial features of these tumors.

## Discussion

NB-FOXR2 is a newly identified and often misdiagnosed entity, which has contributed to the paucity of available data on this tumor subgroup and the lack of faithful preclinical models. In this study, we report that NB-FOXR2 patient tumors transcriptionally mirror *LHX6*^+^/*DLX*^+^ MGE-derived interneurons. They retain a TF fingerprint of their developmental anatomical niche, which is specific to the MGE and its neuronal derivatives. Confirming the potential of this niche as a candidate origin, *in utero* targeting of *Foxr2* and p53 loss of function to the GE produced tumors with high penetrance and transcriptional characteristics of MGE-derived neurons and glia.

Cortical interneurons have many developmental features that could be relevant for the study of these tumors, some of which are specific to humans. A recent study of human GEs (64) identified nests of proliferating *NKX2-1*+ neuroblasts in the MGE persisting until 39 PCW (term birth). This configuration of dividing cells was not observed in the LGE nor in the rodent brain. Interneurons can also be classified into several subtypes based on their morphologies, activity, and neuropeptides; and interneuron progenitors acquire specific interneuron subtype identity during fetal development (38). Furthermore, interneurons are subject to programmed cell death and undergo activity-dependent maturation during postnatal development (65, 66). We noted that many NB-FOXR2 patient tumors express SST, a neuropeptide that characterizes the MGE-derived somatostatin interneuron subtype. Also, in contrast to the typical progenitor-like phenotype observed in many pediatric brain tumors, NB-FOXR2 have a somewhat more differentiated state, as evidenced by the finding of high expression of the later induced *DLX5/6* and low expression of the earlier induced *DLX1/2* (67, 68). Finally, NB-FOXR2 tumors also express OPC lineage TFs OLIG2 and SOX10 but lack expression of PDGFRA, a receptor tyrosine kinase important for OPC specification. Taken together with our findings of Foxr2 binding upstream of key glial genes sufficient for initiating gliogenesis, we propose that aberrant expression of *FOXR2* in an MGE progenitor leads to reprogramming toward a glial cell fate, promoting this indeterminate dual state. Future lineage tracing studies in NB-FOXR2 murine models, potentially in parallel with other oncogenes targeted to the GE *in utero*, could help to determine whether *Foxr2* is causal for the glial phenotype or whether these cell states reflect the competence of the cell of origin to give rise to different lineages.

This study adds to a growing body of evidence for the involvement of interneurons in brain pathologies. NB-FOXR2 tumors are not the only tumor type to be associated to a cortical interneuron lineage; H3.3G34R/V HGGs originate in *GSX2*+ interneuron progenitors, and the chromatin state at the *GSX2* locus in this cell context underlies the vulnerability to PDGFRA alterations (10, 69, 70). However, unlike NB-FOXR2 tumors, H3.3G34R/V tumors exhibit astrocytic instead of oligodendroglial components, express primarily *DLX1/2*, and lack expression of genes associated with differentiated neurons. Also, the expression of *GSX2*, which is predominantly expressed in the LGE, suggests that H3.3G34R/V HGG may originate in derivatives of the LGE (10). Meanwhile, human midgestation CGE interneuron progenitors are the putative cell of origin for tuberous sclerosis complex caused by germline *TSC1/2* mutations, which inhibit mTOR signaling, and are associated with subependymal nodules and subependymal giant cell astrocytoma (71). Thus, it is conceivable that each GE is intrinsically vulnerable to different oncogenic insults or disruptions in normal cell signaling, resulting in different types of brain malignancies characterized by unique driver alterations. Furthermore, these vulnerabilities might be consequences of human-specific developmental features that allow for the enlarged cortex in the human brain, which has implications for future modeling of these diseases.

Resolving a candidate lineage of origin for NB-FOXR2 tumors enabled us to efficiently design novel *in vivo* models for this tumor by targeting the GEs. Previous studies in modeling *FOXR2*-driven oncogenesis have targeted neural populations more broadly, a likely explanation for many of the challenges to recapitulate patient pathologies in these models. A Sleeping Beauty transposon system used in Nestin-cre mice to induce *Pten* and *Tp53* loss resulted in PNET-like tumors expressing synaptophysin and Olig2/Sox10 (72). A genetically engineered mouse model was designed using a *Rosa26*-*Foxr2* knock-in crossed with Nestin-cre mice and *Trp53*-floxed mice, resulting in tumors arising outside the characteristic NB-FOXR2 brain regions, mainly in the olfactory bulb and brainstem (73) but also outside the brain, e.g., in the hind leg. Although these models captured the dual neuronal and oligodendroglial features of NB-FOXR2 tumors, the lack of neuronal subtype specificity and locations of tumor formation likely reflects the broad targeting of Nestin*+* progenitors. Another model of NRAS activation in embryonic OPCs generated malignant brain tumors in zebrafish, which captured the oligodendroglial profile similar to NB-FOXR2 but lacked a neuronal component (74). Finally, in another IUE model, *Foxr2* (together with *Trp53* loss and either wild-type Pdgfra or Pdgfra^D842V^) targeted to the dorsal cortex induced gliomas (7). Altogether, these studies indicate the extent to which cell context determines the oncogenic consequences of FOXR2 activation. Targeting *Foxr2* directly to the ventral embryonic brain, as we did in this study, in turn, produced tumors that recapitulated the location, histology, and the cell lineage context (the GE) of human tumors, with large compartments resembling GE-derived neurons, and some glial representation. Thus, leveraging comprehensive references of the normal brain is a cost-effective strategy to define the tumor cellular context and rationally minimize modeling efforts, which are time- and labor-intensive.

Our model also mimics the driver pathway alterations of NB-FOXR2. Consistent with chr 1q gains leading to *MDM4* overexpression and subsequent p53 suppression in human tumors, we found that *Foxr2* overexpression was tumorigenic in mice only in the context of p53^LOF^. Indeed, mice with *Foxr2* alone led to alterations in neuronal development and low-grade, nonproliferative lesions containing chromosomal abnormalities. Meanwhile, *Foxr2* together with p53 loss produced aggressive tumors and increased proliferation. Hypoactive p53 may therefore be a dependency of *FOXR2*-driven oncogenesis in this cellular context. An additional advantage of the murine tumor model we present in this study is the ability to derive cell lines that can rapidly engraft in syngeneic, immunocompetent mice, enabling the screening of new therapeutics as well as the study of tumor–microenvironment interactions.

Our study has certain limitations. NB-FOXR2 tumors are rare cancers, limiting our sample size. Given the observational nature of patient tumor data sampled at the time of biopsy or autopsy, our data preclude distinguishing between the cell of origin, which acquired the initiating oncogenic event, and the cell of transformation. However, experimental models such as the IUE model can confirm that progenitor cells in the GEs have the capacity to give rise to cortical brain tumors, although we cannot conclude that the GE is the only progenitor niche that can give rise to NB-FOXR2. At present, this model targets the ventral telencephalon nonspecifically, such that any cells in the GE niche could take up the oncogenes, and future work would be needed to target individual progenitor domains.

In conclusion, we show that NB-FOXR2 adds to the growing number of childhood tumor entities that associate with transcriptionally distinct and anatomically and temporally restricted progenitor niches. The heterogeneous origins and molecular profiles across pediatric brain tumors have limited tumor modeling and therapeutic development so far. Whereas systematically targeting progenitors across brain regions and temporal windows *in vivo* could be prohibitive in terms of cost and labor, we show in this study that, as for HGGs (11, 19), leveraging large-scale reference datasets of the developing brain is an efficient approach to direct modeling strategies in specific progenitor niches. Importantly, the engraftable tumor model established in this study enables further investigation of tumor cell–intrinsic properties and tumor–microenvironment interactions. These models can now be used as a platform for high-throughput CRISPR and drug screens to reveal novel selective pharmacologic vulnerabilities in these aggressive tumors.

## Supplementary Material

Supplementary Table 1 Analyses and materials related to murine IUE and orthotopic models.

Supplementary Table 2 Summary of patient samples included in this study, and profiling by RNAseq, scRNAseq, or scMultiome.

Supplementary Table 3 Post-alignment quality control for bulk RNAseq data for pediatric brain tumors.

Supplementary Table 4 Quality control metrics and processing parameters for single-cell data for patient tumors.

Supplementary Table 5 Quality control metrics and processing parameters for single-cell data for murine models.

Supplementary Table 6 Summary of reference datasets for the normal brain and adrenal gland used in this study.

Supplementary Table 7 Reference dataset labels used for cell type annotation of mouse model single-cell datasets.

Supplementary Table 8 Quantification of expression and detection rate (Pct1) for transcription factors in the normal brain reference datasets.

Supplementary Table 9 Cell type specific gene signatures derived from reference datasets, used for enrichment scoring.

Supplementary Table 10 ssGSEA enrichment of cell type specific gene signatures in bulk RNAseq tumors. For each sample, the highest scoring signature is shown.

Supplementary Figure 1 Quality control (QC) metrics and copy-number variation for scRNAseq datasets of NB-FOXR2 patient tumors.

Supplementary Figure 2 Transcription factor (TF) patterning in normal reference datasets.

Supplementary Figure 3 NB-FOXR2 express a transcription factor fingerprint of the medial ganglionic eminence.

Supplementary Figure 4 Bulk RNAseq projections by ssGSEA.

Supplementary Figure 5 NB-FOXR2 tumors transcriptionally resemble interneurons and oligodendrocyte precursor cells.

Supplementary Figure 6 Mouse models transcriptomically recapitulate human NB-FOXR2

Supplementary Figure 7 Orthotopic engraftment of cell lines derived from Foxr2 p53LOF murine model.
