# Supplementary material for: FOXR2 Targets LHX6+/DLX+ Neural Lineages to Drive Central Nervous System Neuroblastoma
Source: Cancer Res. 2024 Nov 4;85(2):231–50. doi: 10.1158/0008-5472.CAN-24-2248 (PMC11733536; doi:10.1158/0008-5472.CAN-24-2248)
Supplement: Supplementary Figure 1 — Quality control (QC) metrics and copy-number variation for scRNAseq datasets of NB-FOXR2 patient tumors. [file can-24-2248_supplementary_figure_1_suppsf1.pdf]

Supplementary Figure 1

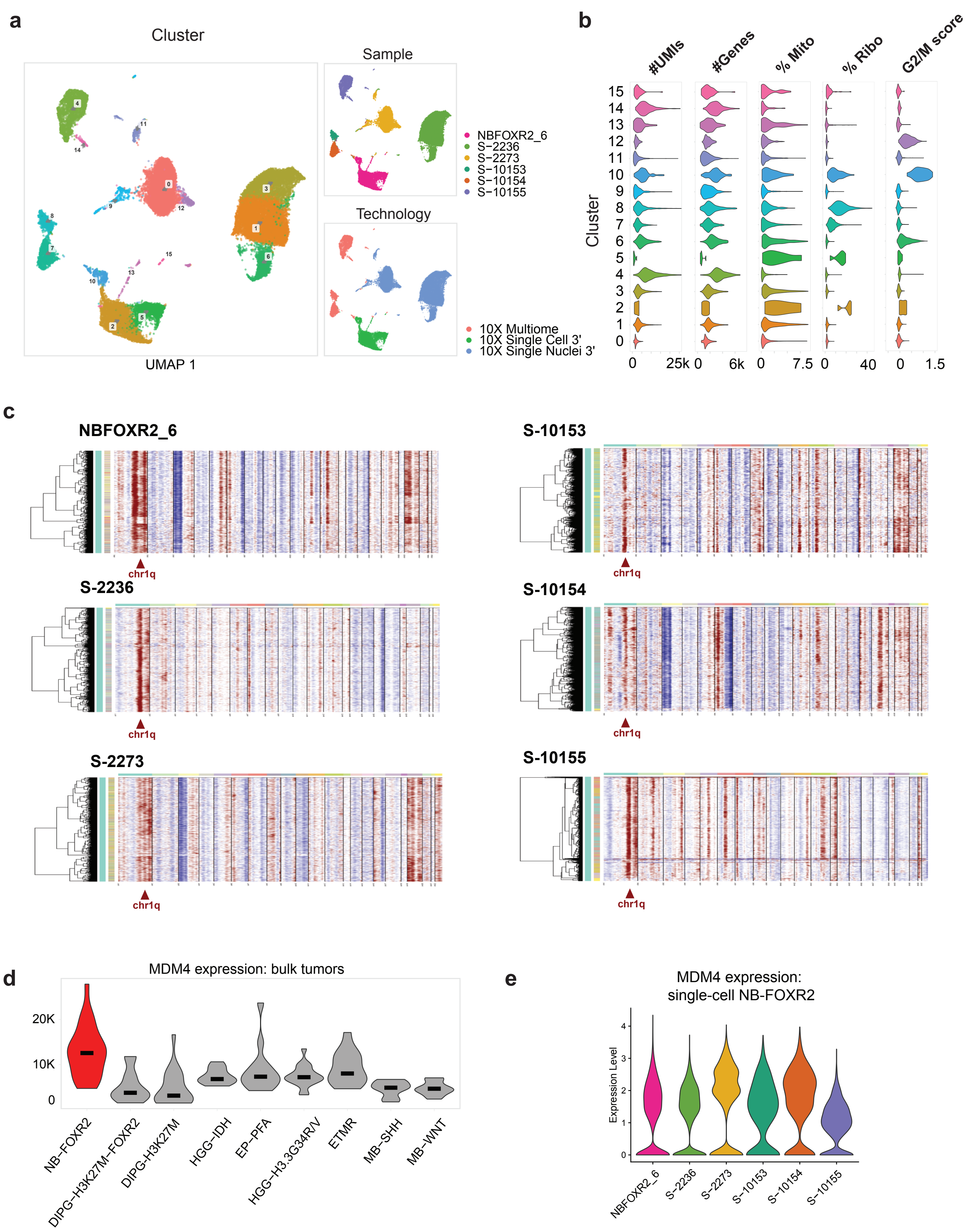

Supplementary Figure 1. Quality control (QC) metrics and copy-number variation for scRNAseq datasets of NB-FOXR2 patient tumors.
a. UMAP joint representation of patient samples, colored by cluster, sample, or technology (without integration or batch correction)
b. QC metrics for joint clusters as in (a). Mito: mitochondrial RNA, Ribo: ribosomal RNA, G2/M: G2 and Mitotic cell cycle phase.
c. Heatmaps showing copy number variation in patient samples computed using InferCNV. Red indicates copy number gain and blue copy number loss.
d. *MDM4* expression across bulk RNAseq tumor samples included in the study cohort.
e. *MDM4* expression across NB-FOXR2 samples profiled with single-cell RNAseq.
