# Supplementary material for: FOXR2 Targets LHX6+/DLX+ Neural Lineages to Drive Central Nervous System Neuroblastoma
Source: Cancer Res. 2024 Nov 4;85(2):231–50. doi: 10.1158/0008-5472.CAN-24-2248 (PMC11733536; doi:10.1158/0008-5472.CAN-24-2248)
Supplement: Supplementary Figure 2 — Transcription factor (TF) patterning in normal reference datasets. [file can-24-2248_supplementary_figure_2_suppsf2.pdf]

Supplementary Figure 2

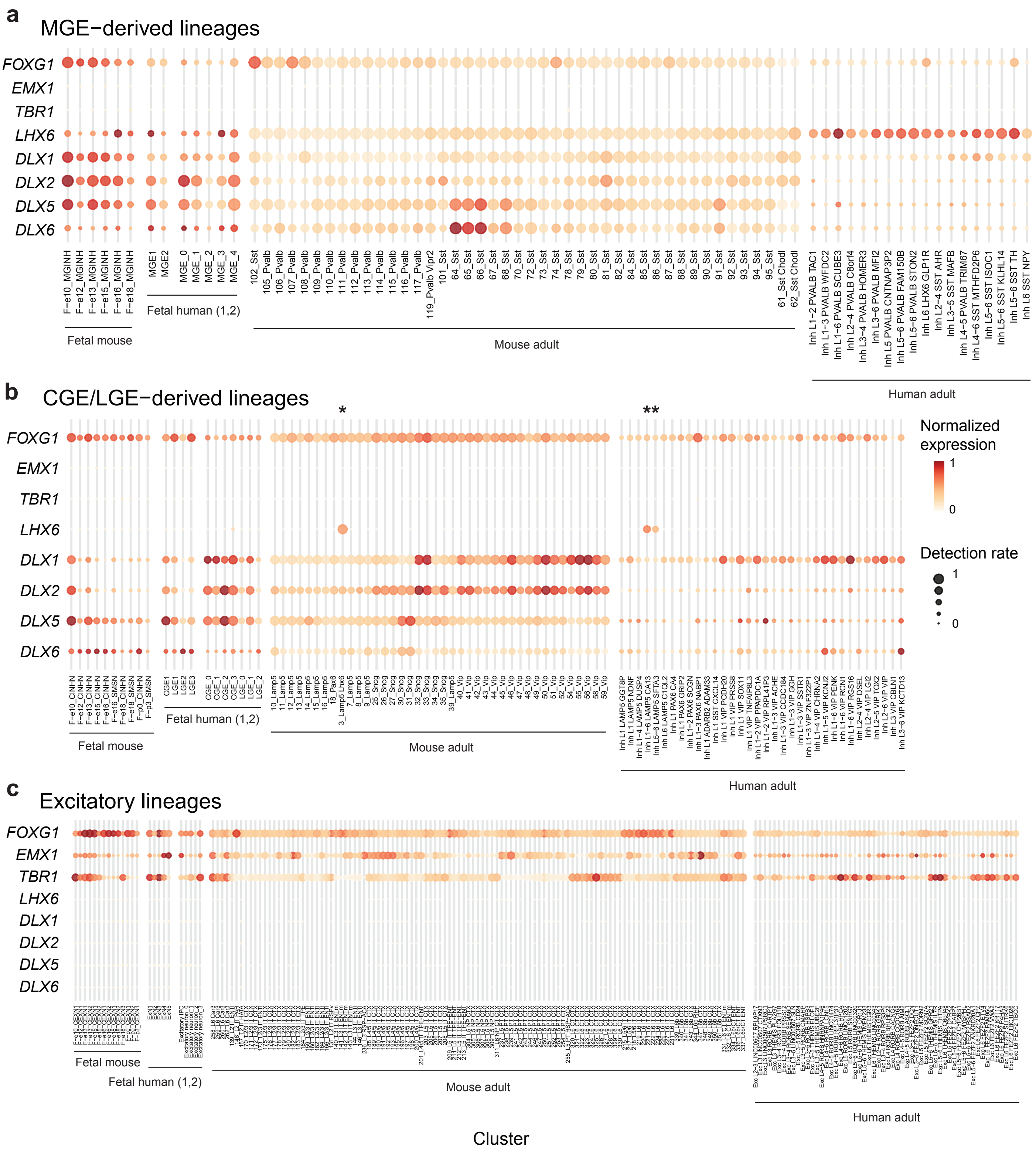

Supplementary Figure 2 (related to Figure 2). Transcription factor (TF) patterning in normal reference datasets.

a-c. Expression of the telencephalon transcription factor (TF) panel across neuronal cell types in the normal brain. Bubble color encodes mean expression across cells, and bubble size encodes detection rate. Asterisks indicate *LHX6*⁺ subtypes of LAMP5 interneurons in adult datasets, which, despite clustering with CGE-derived LAMP5 types, have been suggested to arise in the MGE (Tasic et al, Nature, 2018; Hodge et al, Nature, 2019; Yao et al, Cell, 2021). MGE: medial ganglionic eminence; CGE: caudal ganglionic eminence; LGE: lateral ganglionic eminence.
