# Supplementary material for: FOXR2 Targets LHX6+/DLX+ Neural Lineages to Drive Central Nervous System Neuroblastoma
Source: Cancer Res. 2024 Nov 4;85(2):231–50. doi: 10.1158/0008-5472.CAN-24-2248 (PMC11733536; doi:10.1158/0008-5472.CAN-24-2248)
Supplement: Supplementary Figure 3 — NB-FOXR2 express a transcription factor fingerprint of the medial ganglionic eminence. [file can-24-2248_supplementary_figure_3_suppsf3.pdf]

Supplementary Figure 3

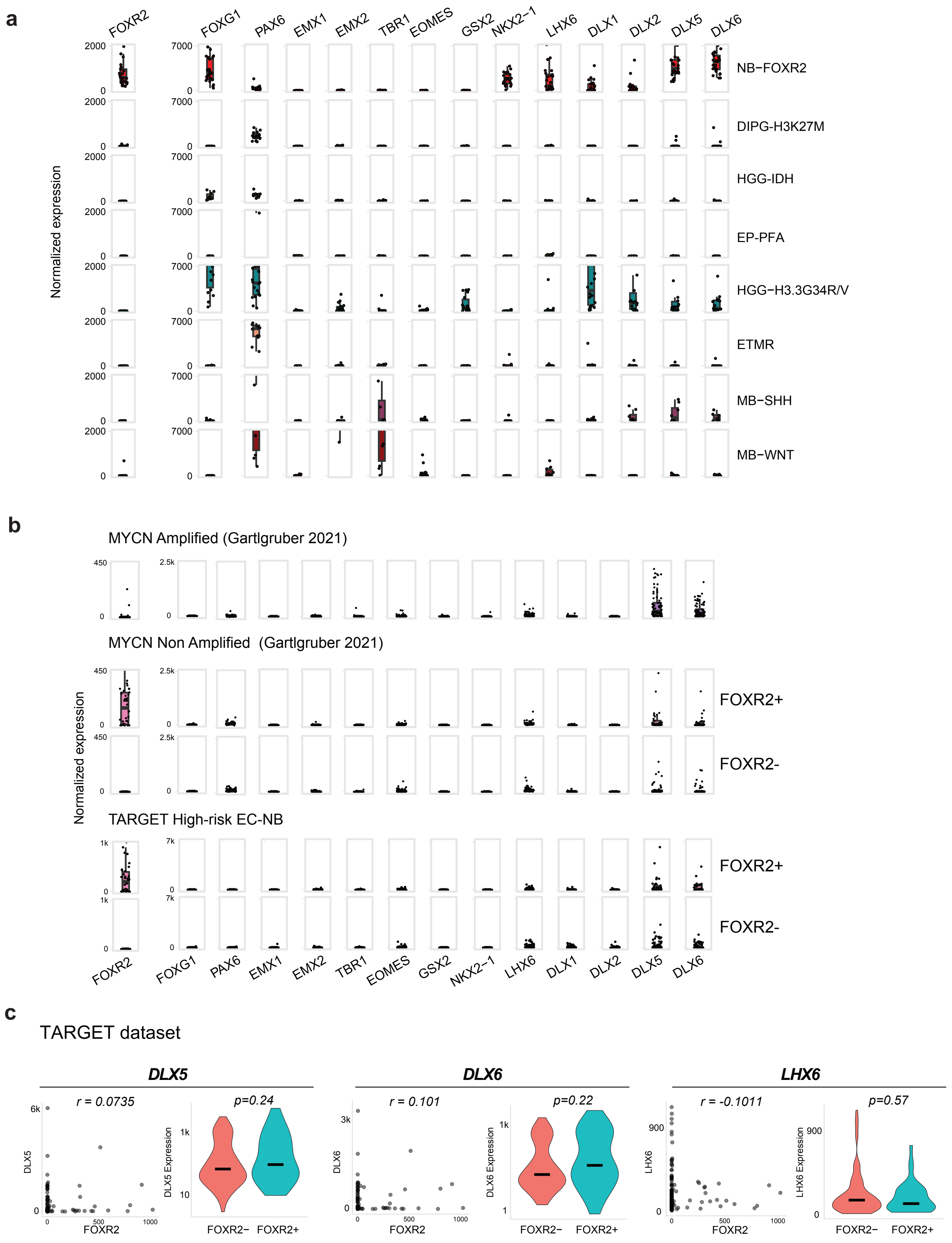

Supplementary Figure 3 (related to Figure 3). NB-FOXR2 express a transcription factor fingerprint of the medial ganglionic eminence.

a. Boxplots of bulk RNAseq expression of telencephalon patterning transcription factors across brain tumors. All plots other than for *FOXR2* share the same y-axis scale.

b. Boxplots of bulk RNAseq of genes from (a) in extra-cranial neuroblastomas. All plots other than for *FOXR2* share the same y-axis scale. *FOXR2*+ tumors: normalized expression > 2.

c. Comparison of TF expression with FOXR2 status in high-risk extracranial neuroblastoma from the TARGET dataset (n=128). Left panels: correspondence between *FOXR2* expression and TF expression, annotated with Pearson correlation. Right panels: TF expression in samples split by *FOXR2* positivity, annotated with p-value (Wilcoxon test). *FOXR2*+ tumors: normalized expression > 2. Y-axis for *DLX5* and *DLX6* violin plots are log10-scaled.
