# Supplementary material for: FOXR2 Targets LHX6+/DLX+ Neural Lineages to Drive Central Nervous System Neuroblastoma
Source: Cancer Res. 2024 Nov 4;85(2):231–50. doi: 10.1158/0008-5472.CAN-24-2248 (PMC11733536; doi:10.1158/0008-5472.CAN-24-2248)
Supplement: Supplementary Figure 4 — Bulk RNAseq projections by ssGSEA. [file can-24-2248_supplementary_figure_4_suppsf4.pdf]

Supplementary Figure 4

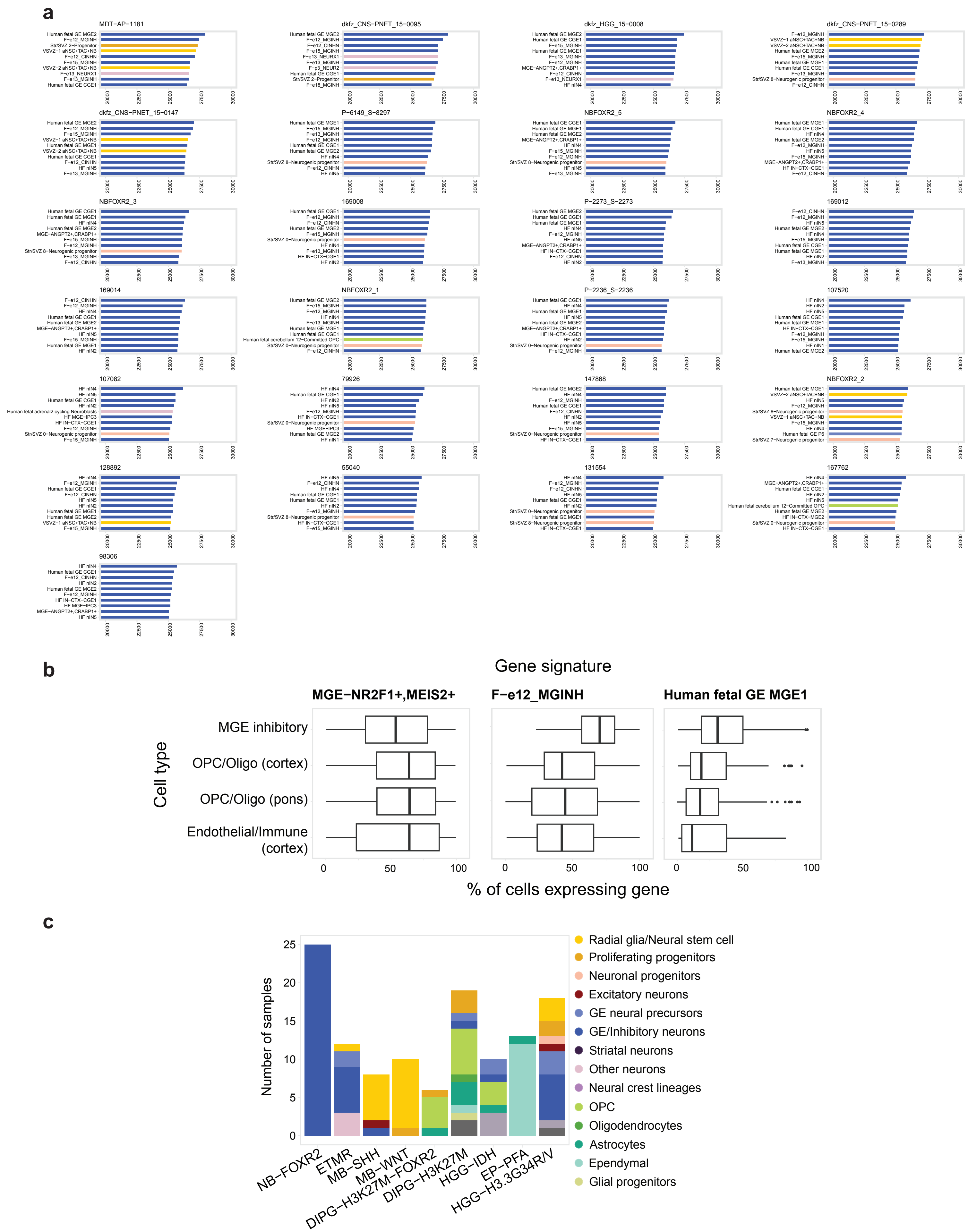

Supplementary Figure 4 (related to Figure 4). Bulk RNAseq projections by ssGSEA.
a. Top 10 scoring signatures by ssGSEA for each NB-FOXR2 bulk tumor sample (N=25); each plot represents one sample. X-axis indicates ssGSEA score.
b. Distribution of detection rate across genes in three MGE gene signatures, in four cell types (used to confirm one signature is not MGE-specific: see Methods). Detection rate is defined as proportion of cells in the cell type where a gene is detected (expression > 0). Each plot represents one gene signature. Cell populations are derived from an atlas of developing murine brain (Jessa et al. Nature Genetics 2022). Broad cell labels (Supplementary Table 7) used to assemble each cell type are as follows. For cells extracted from the cortex, *MGE inhibitory*: “MGE inhibitory neurons”; *OPC/Oligo (cortex)*: “OPC”, “Oligodendrocytes”; *Endothelial/Immune (cortex)*: “Meninges”, “Endothelial”, “Immune”, “Pericytes”. For cells extracted from the pons, *OPC/Oligo (pons)*: “OPC”, “Oligodendrocytes”.
c. Tally of top scoring signature for each bulk pediatric brain tumor sample by ssGSEA.
