# Supplementary material for: FOXR2 Targets LHX6+/DLX+ Neural Lineages to Drive Central Nervous System Neuroblastoma
Source: Cancer Res. 2024 Nov 4;85(2):231–50. doi: 10.1158/0008-5472.CAN-24-2248 (PMC11733536; doi:10.1158/0008-5472.CAN-24-2248)
Supplement: Supplementary Figure 5 — NB-FOXR2 tumors transcriptionally resemble interneurons and oligodendrocyte precursor cells. [file can-24-2248_supplementary_figure_5_suppsf5.pdf]

Supplementary Figure 5

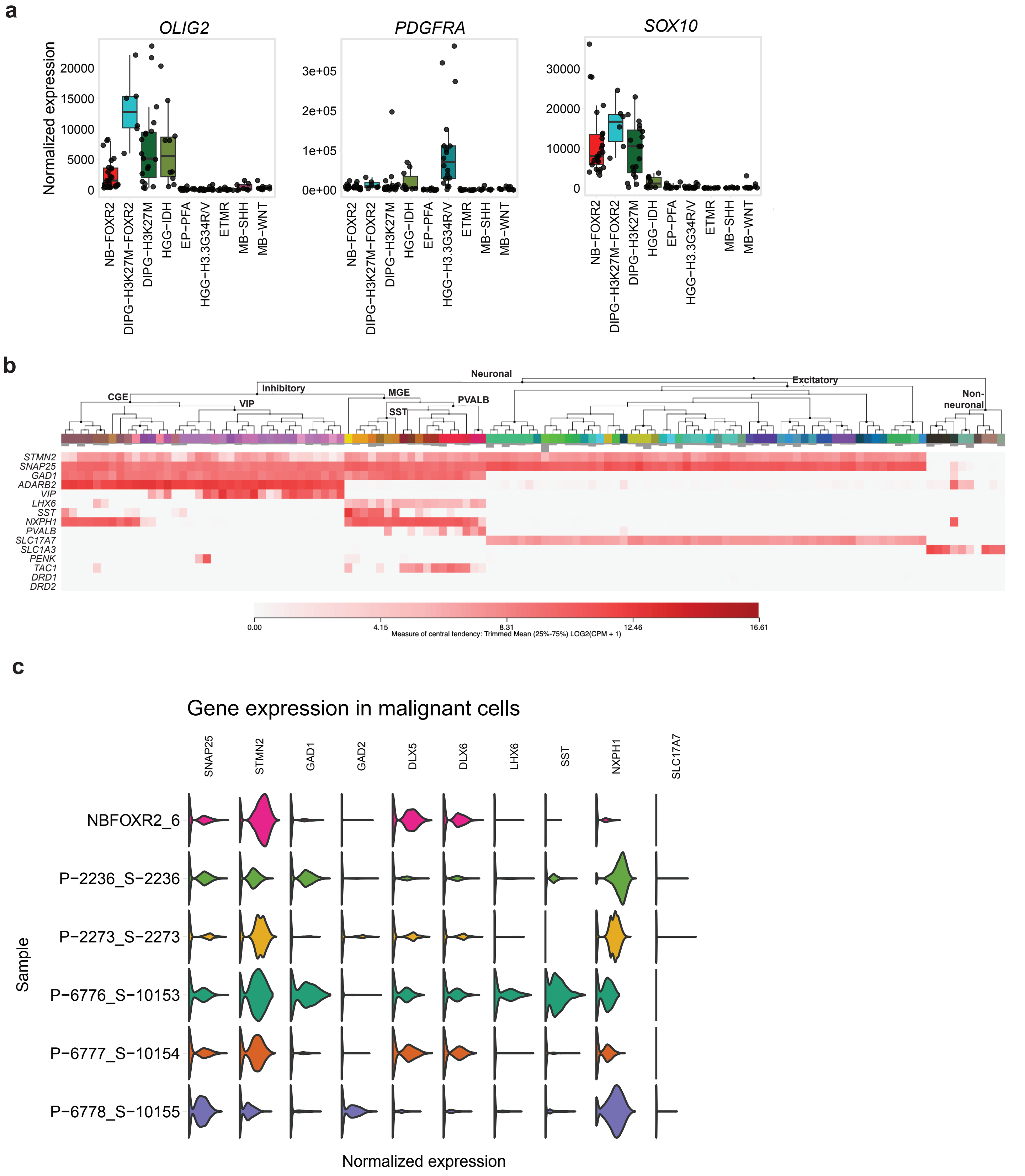

Supplementary Figure 5 (related to Figure 4). NB-FOXR2 tumors transcriptionally resemble interneurons and oligodendrocyte precursor cells.

a. Expression of oligodendroglial gene markers by bulk RNAseq across brain tumor subtypes.

b. Expression of cell type specific canonical marker genes in an atlas of adult human cortex (Hodge et al, Nature, 2019; Allen Brain Atlas).

c. Expression of canonical neuron gene markers in malignant cells of each NB-FOXR2 tumor scRNA-seq dataset (n=6).
