# Supplementary material for: FOXR2 Targets LHX6+/DLX+ Neural Lineages to Drive Central Nervous System Neuroblastoma
Source: Cancer Res. 2024 Nov 4;85(2):231–50. doi: 10.1158/0008-5472.CAN-24-2248 (PMC11733536; doi:10.1158/0008-5472.CAN-24-2248)
Supplement: Supplementary Figure 6 — Mouse models transcriptomically recapitulate human NB-FOXR2 [file can-24-2248_supplementary_figure_6_suppsf6.pdf]

Supplementary Figure 6

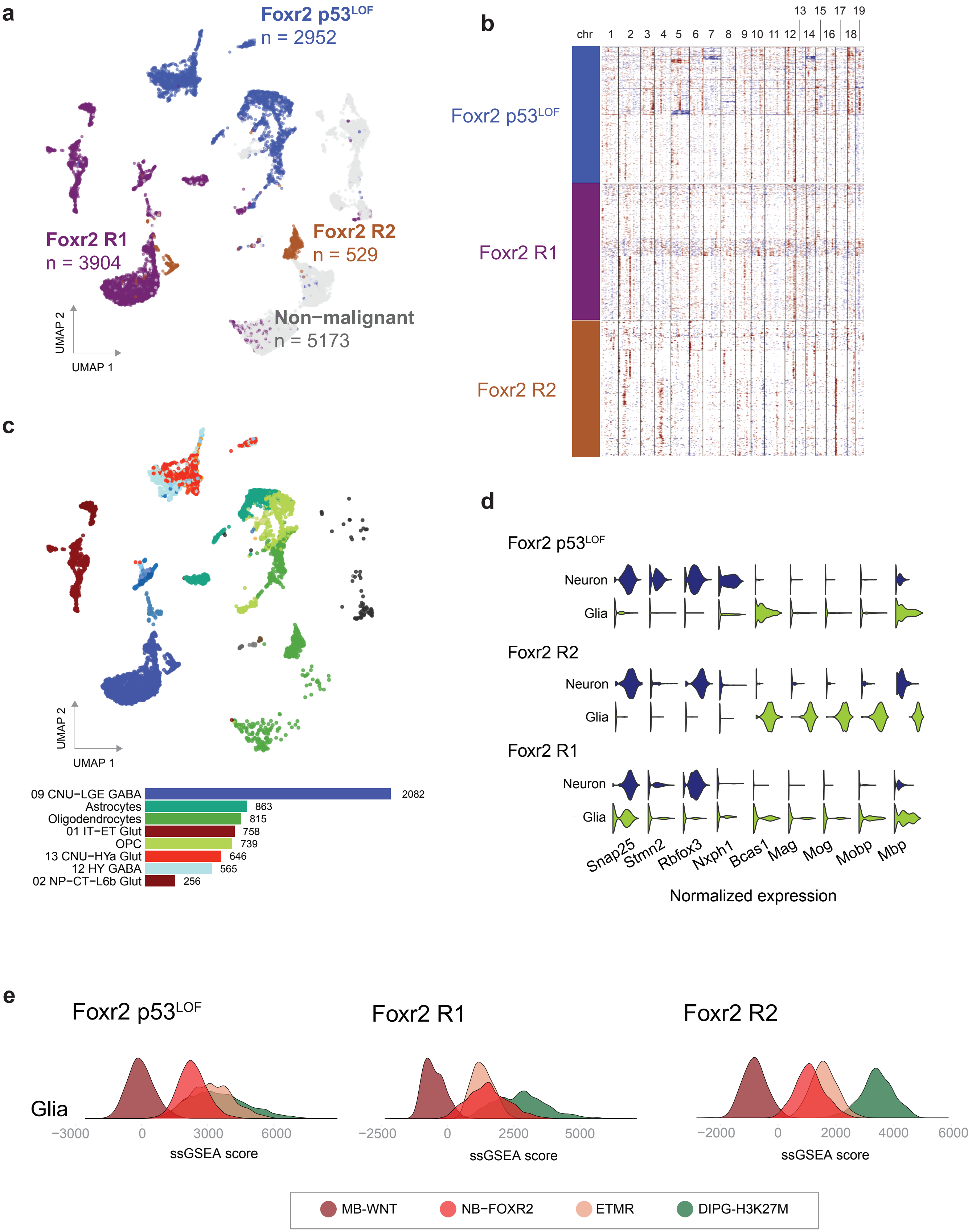

Supplementary Figure 6 (related to Figure 6). Mouse models transcriptomically recapitulate human NB-FOXR2

a. UMAP joint representation of mouse models (n=3) without integration or batch correction. Cells colored by sample, with non-malignant cells colored in grey.

b. Heatmaps of copy number variation for malignant cells in each mouse model, computed with inferCNV.

c. Top: UMAP of malignant cells in mouse models, colored by cell type label resulting from machine learning-based cell type annotation trained with murine forebrain atlases. Cell type color labels correspond to bar plot below. Bottom: Bar plot quantification of number of cells per group in UMAP. Cell types comprising >2% cells in the UMAP are shown.

d. Expression of canonical neuronal and glial gene markers in malignant cells of each mouse model scRNA-seq dataset.

e. Distribution of tumor signature ssGSEA scores in glial-like malignant cells of each mouse model.
