# Supplementary material for: FOXR2 Targets LHX6+/DLX+ Neural Lineages to Drive Central Nervous System Neuroblastoma
Source: Cancer Res. 2024 Nov 4;85(2):231–50. doi: 10.1158/0008-5472.CAN-24-2248 (PMC11733536; doi:10.1158/0008-5472.CAN-24-2248)
Supplement: Supplementary Figure 7 — Orthotopic engraftment of cell lines derived from Foxr2 p53LOF murine model. [file can-24-2248_supplementary_figure_7_suppsf7.pdf]

Supplementary Figure 7

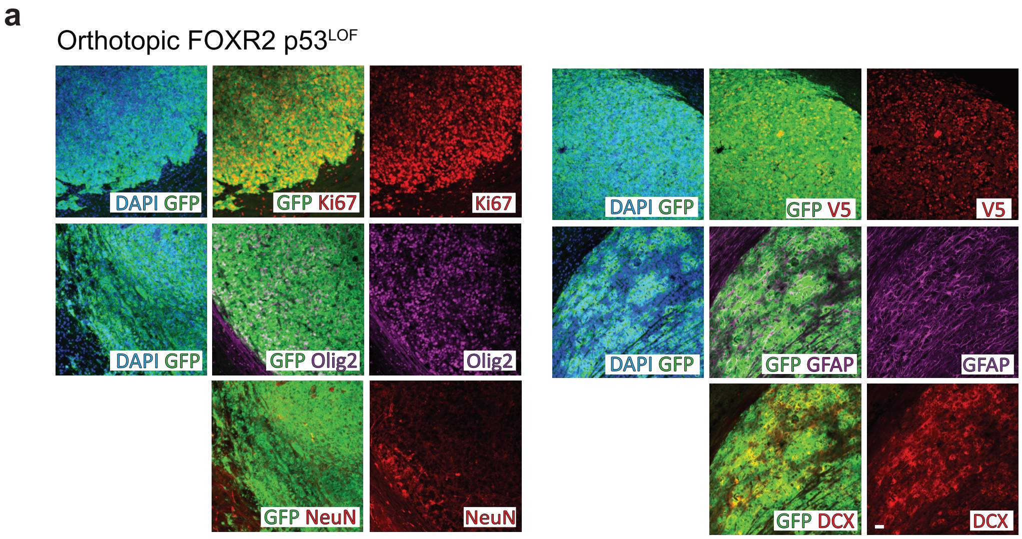

Supplementary Figure 7 (related to Figure 7). Orthotopic engraftment of cell lines derived from Foxr2 p53^{LOF} murine model.

a. Immunofluorescence for GFP, V5, Ki67, Olig2, NeuN, DCX and GFAP in coronal striatal sections from orthotopically engrafted, symptomatic mice. Mice were injected in the striatum with 150,000 FOXR2 p53^{LOF} cells. Tumor cells are GFP+, colocalize with V5, and express high levels of Ki67, Olig2 and DCX. Cells in these lesions express NeuN intermittently, recapitulating the phenotype of the *de novo* models. Scale bar 50μm.
